# Respiratory microbiota of humpback whales may be reduced in diversity and richness the longer they fast

**DOI:** 10.1038/s41598-020-69602-x

**Published:** 2020-07-28

**Authors:** Catharina Vendl, Eve Slavich, Bernd Wemheuer, Tiffanie Nelson, Belinda Ferrari, Torsten Thomas, Tracey Rogers

**Affiliations:** 1 Evolution and Ecology Research Centre, School of Biological, Earth and Environmental Sciences, University of New South Wales, Sydney, NSW 2052 Australia; 2 Stats Central, Mark Wainwright Analytical Centre, School of Mathematics and Statistics, University of New South Wales, Sydney, NSW 2052 Australia; 3 Queensland Facility for Advanced Bioinformatics, Griffith University, Gold Coast, Southport, QLD 4215 Australia; 4 School of Biotechnology and Biomolecular Sciences, University of New South Wales, Sydney, NSW 2052 Australia; 5 Centre for Marine Bio-Innovation, School of Biological, Earth and Environmental Sciences, Sydney, NSW 2052 Australia

**Keywords:** Animal migration, Microbial ecology

## Abstract

Humpback whales endure several months of fasting while undertaking one of the longest annual migrations of any mammal, which depletes the whales’ energy stores and likely compromises their physiological state. Airway microbiota are linked to respiratory health in mammals. To illuminate the dynamics of airway microbiota in a physiologically challenged mammal, we investigated the bacterial communities in the blow of East Australian humpback whales at two stages of their migration: at the beginning (n = 20) and several months into their migration (n = 20), using barcoded tag sequencing of the bacterial 16S rRNA gene. We show that early in the fasting the whale blow samples had a higher diversity and richness combined with a larger number of core taxa and a different bacterial composition than later in the fasting. This study provides some evidence that the rich blow microbiota at the beginning of their fasting might reflect the whales’ uncompromised physiology and that changes in the microbiota occur during the whales’ migration.

## Introduction

The airways of cetaceans^1–7^ and other mammals (humans^8,9^, horses^10^^,^ dogs^11^^,^ cats^12^^,^ mice^13^ harbour a large diversity of bacteria. Studies focusing on airway microbiota of humans^14–17^ and equines^10^ have identified a close relationship between the composition of microbial communities and the airways’ physiological state. A compromised respiratory system typically correlates with an altered microbiota^17^^,^ as it changes the rate of immigration and elimination of bacteria as well as the growth conditions within the airways. The cause that compromises the airways determines the type of change taking place in the microbial communities. In humans, certain conditions like advanced chronic obstructive pulmonary disease (COPD)^18^^,^ chronic rhinosinusitis (CRS) and pneumonia result in a decrease of community richness and in the case of pneumonia also in the dominance of few or even a single opportunistic pathogen, like *Staphylococcus aureus* or *Pseudomonas aeruginosa*^19^. In contrast, other conditions like asthma typically cause an increase in bacterial diversity^20^. Dickson et al.^17^ summarised these points by adapting a famous Tolstoy quote: ‘All healthy lungs are alike,every unhealthy lung is unhealthy in its own way’. Dickson et al.^21^ and Dickson et al.^17^ have suggested a model, characterizing the interplay between respiratory microbiota and their host not only as dynamic and continuous, but also as bidirectional. In other words, any changes taking place within the respiratory system are capable of activating the so called dysbiosis-inflammation cycle leading to shifts of both immune response and microbiota.

Several studies investigating the airway microbiota of whales^2,3,7^ and dolphins^1,4–6,22^ analysed their exhaled breath condensate or ‘blow’. Unlike the studies on humans, the cetacean-focused studies exclusively characterized the blow microbiota of the studied specimens and did not investigate a potential correlation of blow microbiota and overall health. While this would be possible for captive cetaceans, it is often difficult to gain reliable health parameters on whales and dolphins in their natural habitat^2,23^. To gain insight into the dynamics of cetacean airway microbiota in response to physiological challenges, we focused here on East Australian humpback whales (HW), *Megaptera novaeangliae,* (breeding population E1 as defined by the International Whaling Commission^24^ during their annual migration.

**Figure 1:**
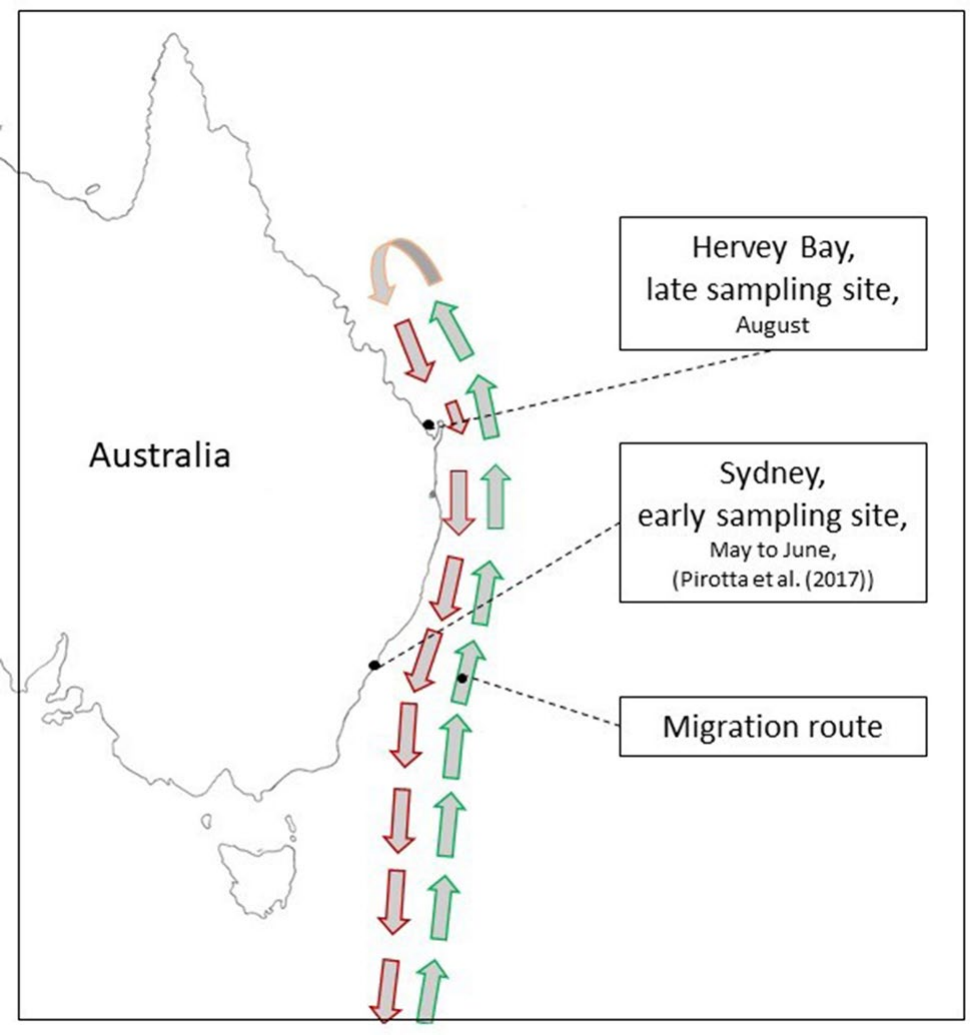
Map of the sampling sites off the coast of Sydney and Hervey Bay, Australia. The green arrows represent the northern leg and the red arrows the southern leg of the annual migration of the East Australian humpback whales. We drew this figure according to the information provided in Franklin^32^.

With a journey that can exceed 8000 km, HW undertake the longest seasonal migration of all mammals^25–28^. The East Australian HW leave their feeding grounds in Antarctica at the end of the austral summer to travel north along the East Australian Coast, until they reach their low latitude breeding and mating areas on the Great Barrier Reef^29^ (Fig. 1). In late winter, they return south to feed in the Southern Ocean. Chittleborough et al.^29^ have reported that the whales’ food intake is marginal during the breeding season, thus resulting in a period of fasting of at least 4 months per year. In addition to the energy requirements for the migration, female HW also spend massive resources on gestation and lactation^30^^,^ whereas males invest large amounts of energy in competing for females^31^.

We hypothesise that an extended period of fasting compromises the whales’ physiological state, which results in a change of the microbial community composition of the airways. We therefore compared the blow microbiota of HW at the beginning of their northern migration along the East Australian Coast (HumpbackNM), where they were at the beginning of their fasting, to those after approximately 3–4 months of fasting during the southern leg of their migration (HumpbackSM).

## Results

### Dataset overview

We collected 20 blow samples from HW, seven seawater (SeawaterSM, environmental controls) and six air (technical controls) samples in Hervey Bay, Southern Queensland, Australia, during August 2017. The HW enter Hervey Bay on the southern leg of their migration a few weeks after leaving their breeding grounds at the Great Barrier Reef and 3–4 months after leaving^33,34^ their feeding grounds in Antarctica. We compared their blow microbiota based on 16S rRNA gene sequencing with the blow microbiota from 20 different individuals in the same population of HW along with six air samples collected by Pirotta et al.^3^off the coast of Sydney, New South Wales, Australia, during May and June 2017, about 1 month after the whales left their feeding grounds in Antarctica, and 26 seawater samples (SeawaterNM) obtained from the same area as Pirotta’s HumpbackNM samples^35^.

We detected a total of 11,573,157 raw 16S rRNA gene sequences, which were clustered into 8838 zero-distance operational taxonomic units (zOTUs). We removed 336 zOTUs as they had an overall relative abundance of less than 0.0001% and deleted 465 zOTUs from the dataset, which were present and highly abundant in most air samples (technical controls). The resulting dataset of whale and seawater samples contained 8,037 zOTUs with a mean of 78,827 reads per sample (sd = 116,046). The rarefaction curves (Supplementary Fig S1) and Good’s coverage (HW–NM: 99.9398, sd = 0.0365; SeawaterNM: 98.7193, sd = 0.5321; HW–SM: 99.9346, sd = 0.0348; SeawaterSM: 99.7689, sd = 0.02747) after filtering showed that the majority of samples was sequenced to near-saturation. The 8,037 zOTUs covered 681 genera, 295 families, 162 orders, 63 classes and 30 phyla.

### Whales at the beginning of their fasting had a higher alpha diversity than the whales at a later stage

We calculated richness, Shannon–Wiener diversity index, Chao1^36,37^ and ACE^38,39^ species estimator for whale and seawater samples using rarefied counts (lowest number of reads: 3432) to account for the difference in sampling depth (Table 1). SeawaterNM and SeawaterSM did not significantly differ in their richness (Kruskal–Wallis test with Holm’s adjustment for multiple comparisons^40,41^: Z = 1.9394, *p* value = 0.0524), Shannon–Wiener diversity (Z = 1.5897, *p* value = 0.1119), Chao1 (Z = 1.7819, *p* value = 0.0748) and ACE species estimator (Z = 1.8287, *p* value = 0.0674). The seawater samples had a significantly higher richness, Chao1 and ACE than their corresponding whale samples (richness: SeawaterNM–HumpbackNM: Z = − 3.7093, *p* value = 0.0005; SeawaterSM–HumpbackSM: Z = − 3.1001, *p* value = 0.0029; Chao1: SeawaterNM–HumpbackNM: Z = − 4.5099, *p* value < 0.0000; SeawaterSM–HumpbackSM: Z = − 3.3819, *p* value = 0.0014; ACE: SeawaterNM–HumpbackNM: Z = − 4.2640, *p* value < 0.0000; SeawaterSM–HumpbackSM: Z = − 3.2611, *p* value = 0.0022). Interestingly, the HumpbackSM samples showed a lower richness, Chao1 and ACE than HumpbackNM samples (richness: Z = 3.428, *p* value = 0.0012; Chao1: Z = 2.8542, *p* value = 0.0065; ACE: Z = 2.9809, *p* value = 0.0043). Also, the Shannon Wiener diversity of HumpbackSM samples was significantly lower than HumpbackNM samples (Z = 5.6562, *p* value < 0.0000) (Table 1). The entire results of the Kruskal–Wallis tests of alpha diversity parameters are displayed in Table 2 and Supplementary Table S1. We also calculated and compared the same alpha diversity values with unrarefied counts (Supplementary Table S2) and received very similar results (Supplementary Table S1).

**Table 1:** Number of reads and alpha diversity parameters (samples size, richness, Shannon index, Chao1 and ACE) of rarefied data (rarefied number of reads = 3432) of samples of 20 humpback whale (HumpbackNM) and 26 seawater (SeawaterNM) samples at the beginning of their fasting and of 20 humpback whale (HumpbackSM) and 7 seawater (SeawaterSM) samples at a later stage. Numbers are after deleting putative technical contaminant zOTUs from the dataset.

Species	Sample size (n)	Reads per sample, mean (Sd)	Richness (number of OTUs per sample) mean (sd)	Shannon index (sd)	Chao1 species estimator (sd)	ACE species estimator (sd)
HumpbackNM	20	232,201 (143,670)	454 (228)	5.38 (0.64)	495 (263)	537 (289)
HumpbackSM	20	21,091 (21,843)	86 (114)	3.32 (0.81)	119 (126)	109 (125)
SeawaterNM	26	40,593 (13,628)	755 (111)	5.50 (0.22)	996 (181)	1163 (201)
SeawaterSM	7	80,761 (8,648)	604 (25)	5.25 (0.10)	730 (37)	832 (28)

**Table 2:** Z and *p* values of Kruskal–Wallis test of richness, Shannon–Wiener diversity, Chao1 and ACE species estimator of rarefied counts. The upper value is the Z, the lower the *p *value in each cell. Those *p* values with an asterisk are statistically significant.

	HumpbackNM	HumpbackSM	SeawaterNM
**Richness**	Z	Z	Z
	*p* value	*p* value	*p* value
HumpbackSM	3.4280		
	0.0012*		
SeawaterNM	− 3.7093	− 7.3540	
	0.0005*	0.0000*	
SeawaterSM	− 0.6317	− 3.1001	1.9394
	0.2638	0.0029*	0.0524
**Diversity**			
HumpbackSM	5.6562		
	0.0000*		
SeawaterNM	0.2017	− 5.8120	
	0.4201	0.0000*	
SeawaterSM	1.6781	− 2.3949	1.5897
	0.1400	0.0333	0.1119
**Chao1**			
HumpbackSM	2.8542		
	0.0065*		
SeawaterNM	− 4.5099	− 7.5446	
	0.0000*	0.0000*	
SeawaterSM	− 1.3266	− 3.3819	1.7819
	0.0923	0.0014*	0.0748
**ACE**			
HumpbackSM	2.9809		
	0.0043*		
SeawaterNM	− 4.2640	− 7.4333	
	0.0001*	0.0000*	
SeawaterSM	− 1.1146	− 3.2611	1.8288
	0.1325	0.0022*	0.0674

**Figure 2:**
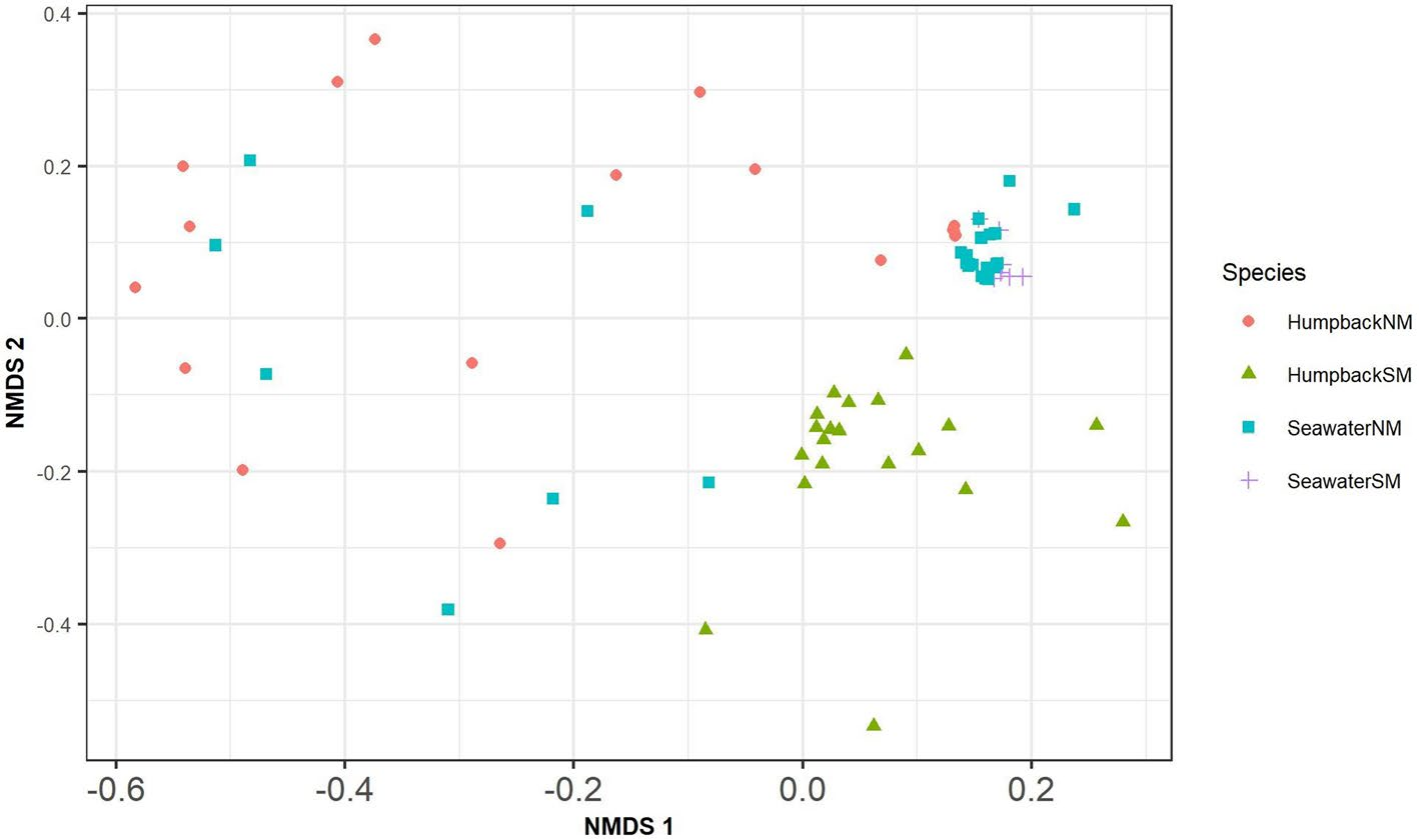
nMDS of microbiota, based on Bray–Curtis dissimilarity and unrarefied data, found in the blow of 20 humpback whale (HumpbackNM) and 26 seawater (SeawaterNM) samples at the beginning of the whales’ fasting and of 20 humpback whale (HumpbackSM) and 7 seawater (SeawaterSM) samples at a later state after deleting putative technical contaminant zOTUs from the dataset. The nMDS plot shows group-specific clustering of HumpbackSM, SeawaterSM and SeawaterNM, whereas the HumpbackNM samples did not form a distinct cluster. An overlap between whale and seawater samples, especially in the case of HumpbackNM and HumpbackNM is observed.

**Figure 3:**
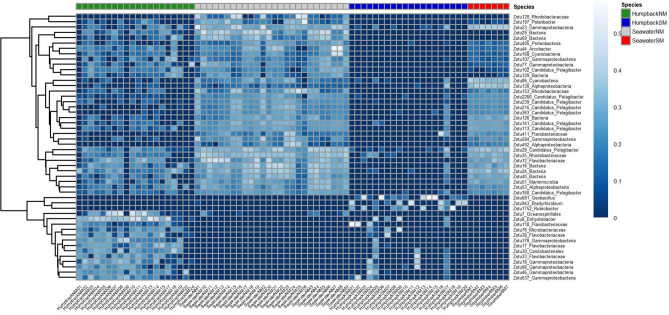
Heatmap of fourth-root transformed relative abundance of the 50 most abundant zOTUs that were identified as significantly different when comparing the microbiota in the blow of humpback whales at the beginning of their fasting (HumpbackNM) and humpback whales at a later stage of fasting (HumpbackSM) and their corresponding seawater samples (SeawaterNM, SeawaterSM).

**Figure 4:**
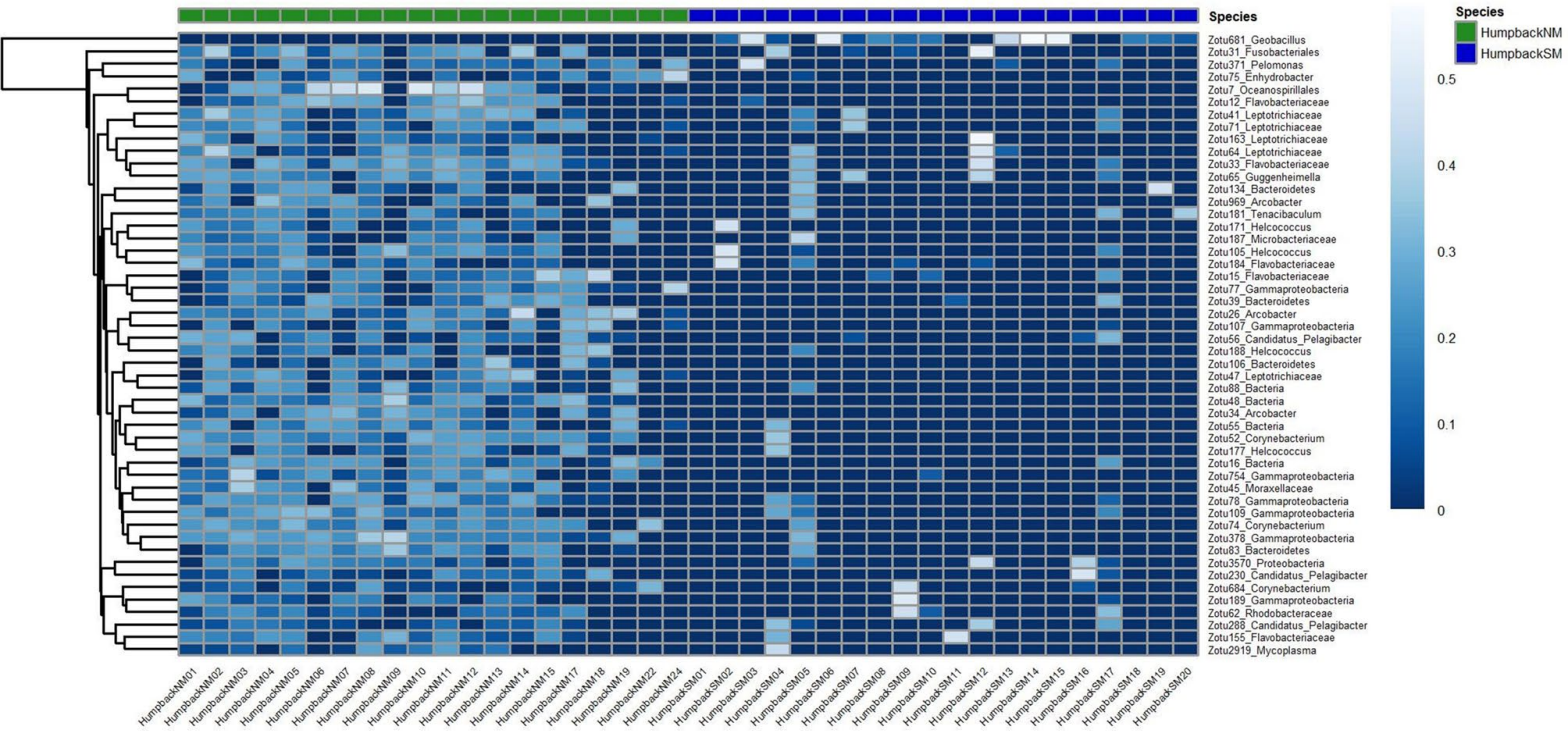
Heatmap of fourth-root transformed relative abundance of the 50 most abundant (out of 311) zOTUs that were identified as significantly different when comparing the microbiota in the blow of humpback whales at the beginning of their fasting (HumpbackNM) and humpback whales at a later stages of fasting (HumpbackSM).

The beta diversity analysis based on the Bray–Curtis dissimilarity coefficient of unrarefied relative abundances and ordinated in a non-metric multidimensional scaling (NMDS) plot (Fig. 2) showed group-specific and relatively tight clustering of the HumpbackSM, SeawaterSM and SeawaterNM replicates, whereas the replicates of HumpbackNM samples did not form a distinct cluster. An overlap between whale blow and seawater samples, especially in the case of HumpbackNM and SeawaterNM was observed. The PCoA plot of the weighted and unweighted generalized UNIFRAC distances (Supplementary Figures S2 and S3) displayed a tighter clustering of the HumpbackNM samples, whereas the HumpbackSM samples were more spread out. To determine if the composition of bacterial communities was significantly different between the four groups (SeawaterNM, HumpbackNM, SeawaterSM, HumpbackSM), we fitted negative binomial models to each zOTU. We detected a significant difference between the bacterial communities of HumpbackNM and HumpbackSM (sum-of-LR = 16,626, *p* = 0.001), of HumpbackSM and SeawaterSM (sum-of-LR = 32,792, *p* = 0.001), of HumpbackNM and SeawaterNM (sum-of-LR = 86,687, *p* = 0.001) and of SeawaterNM and SeawaterSM (sum-of-LR = 35,528, *p* = 0.001). The negative binomial models also identified those zOTUs that accounted for the majority of differences in the microbiota across the samples of HumpbackSM, HumpbackNM, SeawaterSM and SeawaterNM. The heatmap in Fig. 3 shows the prevalence of the most abundant 50 of those zOTUs. When applying the negative binomial models to the zOTUs of the blow samples of HumpbackSM and HumpbackNM only, we identified 311 zOTUs as significantly different between the two whale groups (Fig. 4). These zOTUs accounted for 15% (HumpbackSM) and 33% (HumpbackNM) of total relative sequence abundance and mostly belonged to unclassified genera. The first and second most abundant genera that could be classified were Corynebacterium (HumpbackSM: 1%; HumpbackNM: 2%) and Helcococcus (HumpbackSM: 1%; HumpbackNM: 2%).

### Whales at the beginning of their fasting had a diverse and abundant core

Another useful tool to determine a change in microbial community composition across hosts or time is the identification of core taxa^42–46^. We used an 80% threshold (taxa present in 80% of all sampled individuals) to determine the core microbiota within the blow of HumpbackNM and HumpbackSM samples at two taxonomic levels (zOTU and genus) (Tables 3, 4). To apply a conservative estimate of core we excluded those zOTUs that were commonly found in seawater, as we were unable to tell if these were contaminants of the blow samples or true residents of the airways. The HumpbackNM samples contained 55 core zOTUs that accounted for a total relative read abundance of 14%. 13 zOTUs were disregarded as core due to their common association with seawater. In contrast, the HumpbackSM samples did not share a single core zOTU that was not also present in seawater. The large majority of core zOTUs (42 out of 55) of HumpbackNM were most similar to 16S rRNA gene sequences detected in the mouth of bottlenose dolphins (e.g. GenBank accession: KC258936.1)^1^ (Table 1). Out of these zOTUs, 27 belonged to the class *Gammaproteobacteria* and more precise classification was not possible for most of these sequences. Another 13 core zOTUs of HumpbackNM were similar to sequences found on skin and nares (KF104811.1) and in the mouth of humans and other mammals (JN713454.1). The 42 dolphin-related zOTUs accounted for a total relative abundance of 12%. The HumpbackNM samples had 20 core genera with a total relative abundance of 18%, whereas HumpbackSM had only one belonging to the genus *Geobacillus,* accounting for 5% (Table 4). The most abundant core genera of HumpbackNM with relative read abundances of more than 1.5% each were *Arcobacter, Corynebacterium, Enhydrobacter, Helcococcus and Tenacibaculum*. The two groups of HW did not share any core genera. The findings that HumpbackNM had a large number of core zOTUs and genera and therefore overall more diverse blow microbiota than HumpbackSM was also observed at class level (Supplementary Fig S4).

**Table 3:** Taxonomy, relative abundance and details of environment of core zOTUs in the blow of 20 humpback whales at the beginning of their fasting (HW–NM) and 20 humpback whales at a later stage (HW–SM), listed in descending order of the zOTUs’ relative abundance. An 80% threshold (core taxa present in 80 of individuals in each group) was used. Whereas HumpbackNM samples showed a large number of core zOTUs, HumpbackSM had none that were not associated with seawater.

zOTU no	Taxonomic affiliation	Rel.abund. in HW–SM	Rel.abund. in HW–NM	Environment of most similar sequences	Genbank accession
Zotu8	*Enhydrobacter*		0.0230	Bottlenose dolphin mouth	JQ216650.1
Zotu17	*Flavobacteriaceae*		0.0060	Bottlenose dolphin mouth	KC259203.1
Zotu18	*Gammaproteobacteria*		0.0060	Bottlenose dolphin mouth	JQ216650.1
Zotu30	*Cardiobacteriales*		0.0047	Bottlenose dolphin mouth	JQ216650.1
Zotu33	*Flavobacteriaceae*		0.0047	Bottlenose dolphin mouth	KC259203.1
Zotu26	*Arcobacter*		0.0047	Bottlenose dolphin mouth	KC259203.1
Zotu38	*Flavobacteriaceae*		0.0044	Bottlenose dolphin mouth	KC259203.1
Zotu32	*Gammaproteobacteria*		0.0043	Bottlenose dolphin mouth	JQ216650.1
Zotu378	*Gammaproteobacteria*		0.0043	Bottlenose dolphin mouth	KC259203.1
Zotu80	*Enhydrobacter*		0.0035	Bottlenose dolphin mouth	KC259203.1
Zotu46	*Gammaproteobacteria*		0.0034	Bottlenose dolphin mouth	KC259203.1
Zotu48	*Bacteria*		0.0034	Bottlenose dolphin mouth	JQ216650.1
Zotu37	*Sphingomonas*		0.0033	Human nares and skin	KF100134.1
Zotu3196	*Enhydrobacter*		0.0032	Bottlenose dolphin mouth	JQ216749.1
Zotu637	*Gammaproteobacteria*		0.0030	Bottlenose dolphin mouth	JQ193450.1
Zotu52	*Flavobacteriaceae*		0.0029	Human skin	KF102327.1
Zotu78	*Gammaproteobacteria*		0.0027	Bottlenose dolphin mouth	KC259203.1
Zotu74	*Corynebacterium*		0.0026	Horse skin	CP012136.1
Zotu61	*Gammaproteobacteria*		0.0026	Bottlenose dolphin mouth	KC258450.1
Zotu1300	*Gammaproteobacteria*		0.0024	Bottlenose dolphin mouth	JQ216650.1
Zotu76	*Microbacteriaceae*		0.0024	Human skin	JQ259576.1
Zotu122	*Bacteria*		0.0023	Bottlenose dolphin mouth	KC259203.1
Zotu101	*Leptotrichiaceae*		0.0023	Bottlenose dolphin mouth	KC259203.1
Zotu2000	*Bacteroidetes*		0.0022	Bottlenose dolphin mouth	JQ216650.1
Zotu142	*Gammaproteobacteria*		0.0018	Bottlenose dolphin mouth	KC258936.1
Zotu108	*Gammaproteobacteria*		0.0018	Bottlenose dolphin mouth	KC259203.1
Zotu111	*Microbacterium*		0.0018	Bottlenose dolphin mouth	KC258936.1
Zotu1128	*Gammaproteobacteria*		0.0017	Bottlenose dolphin mouth	JQ216650.1
Zotu125	*Gammaproteobacteria*		0.0016	Bottlenose dolphin mouth	KC259203.1
Zotu157	*Candidatus_Pelagibacter*		0.0016	Bottlenose dolphin mouth	JQ212195.1
Zotu159	*Corynebacteriaceae*		0.0016	Human skin	JF181360.1
Zotu2138	*Gammaproteobacteria*		0.0016	Bottlenose dolphin mouth	KC259203.1
Zotu176	*Microbacteriaceae*		0.0015	Mammal skin, mouth	GQ358839.1
Zotu174	*Corynebacterium*		0.0015	Human skin	JF150986.1
Zotu162	*Gammaproteobacteria*		0.0015	Bottlenose dolphin mouth	KC259203.1
Zotu118	*Flavobacteriaceae*		0.0014	Bottlenose dolphin mouth	JQ216650.1
Zotu139	*Escherichia coli*		0.0014	Mammal gut	CP024997.2
Zotu191	*Leucobacter sp.*		0.0013	Mammal mouth	JN713454.1
Zotu524	*Corynebacterium*		0.0013	Human and pig skin	CP021417.1
Zotu203	*Gammaproteobacteria*		0.0013	Bottlenose dolphin mouth	JQ216650.1
Zotu510	*Bacteroidetes*		0.0012	Bottlenose dolphin mouth	JQ216650.1
Zotu305	*Fusobacterium*		0.0012	Bottlenose dolphin mouth	KC259203.1
Zotu319	*Bacteroidetes*		0.0010	Bottlenose dolphin mouth	KC259203.1
Zotu242	*Cardiobacteriales*		0.0010	Bottlenose dolphin mouth	JQ215966.1
Zotu332	*Corynebacterium*		0.0009	Human, bovine skin	KF104811.1
Zotu744	*Corynebacterium*		0.0009	Human, animal skin	JN834210.1
Zotu315	*Moraxellaceae*		0.0009	Bottlenose dolphin mouth	KC259203.1
Zotu398	Rhodobacteraceae		0.0009	Bottlenose dolphin mouth	KC259203.1
Zotu341	Corynebacterium		0.0009	Human nares	HM265718.1
Zotu604	Gammaproteobacteria		0.0005	Bottlenose dolphin mouth	KC259203.1
Zotu840	Gammaproteobacteria		0.0005	Bottlenose dolphin mouth	KC259203.1
Zotu2302	Cardiobacteriales		0.0004	Bottlenose dolphin mouth	KC259203.1
Zotu1208	Pseudomonadales		0.0003	Bottlenose dolphin mouth	KC258984.1
Zotu2648	Enhydrobacter		0.0002	Bottlenose dolphin mouth	KC260796.1
Zotu4075	Firmicutes		0.0001	Bottlenose dolphin mouth	JQ209287.1

**Table 4:** Taxonomy and relative abundance of core genera of the blow of 20 humpback whales at the beginning of their fasting (HW–NM) and 20 humpback whales at a later stage (HW–SM) in descending order of the genera’s relative abundance. An 80% threshold (core taxa present in 80 of individuals in each group) was used. Whereas the HumpbackNM samples showed a large number of core genera, the HumpbackSM samples had only one.

Core genus	Rel.abund. in HW–SM	Mean/median rel. abund. in HW–SM	Rel.abund. in HW–NM	Mean/median rel. abund. in HW–NM
Geobacillus	0.0477	0.0030/0.0006		0.0009/0.0001
Enhydrobacter			0.0360	
Corynebacterium			0.0277	
Arcobacter			0.0256	
Helcococcus			0.0255	
Tenacibaculum			0.0186	
Acinetobacter			0.0074	
Streptococcus			0.0060	
Guggenheimella			0.0053	
Mycoplasma			0.0043	
Treponema			0.0042	
Staphylococcus			0.0040	
Phocoenobacter			0.0039	
Gulosibacter			0.0033	
Fusobacterium			0.0032	
Pseudomonas			0.0030	
Psychrobacter			0.0021	
Escherichia/Shigella			0.0018	
Sphingomonas			0.0016	
Nocardioides			0.0011	

In addition, we determined those zOTUs and genera as ‘overall’ core that were shared across at least 80% of all 40 whale blow samples (HumpbackNM and HumpbackSM). The only zOTU matching these criteria was seawater-associated and therefore disregarded. Hence, the 40 whale blow samples did not share any overall core zOTUs. The same applied to the genera. Whereas *Pseudomonas* and *Corynebacterium* were shared by 29 (73%) and 28 (70%) whale blow samples, respectively, none of the genera were harboured by 80%.

### Potential marine mammal-specific pathogens in HW blow

In the blow of the HW at the late stage of their migration, we found eight out of a total of 146 genera that were previously identified as pathogens in marine mammals. These eight genera accounted for a total relative abundance of 7%. The HW early in their migration harboured ten potential marine mammal pathogens that included those found in their conspecifics later in their migration. These ten genera out of a total of 294 accounted for a relative abundance of 6%. The potentially pathogenic genera shared by both whale groups included *Corynebacterium, Pseudomonas, Staphylococcus, Fusobacterium, Mycoplasma, Streptococcus, Acinetobacter*, *Stenotrophomonas*^47–49^. Table 5 lists the medical conditions caused by these genera and the affected species.

**Table 5:** Potential marine mammal-specific pathogenic genera found in the blow of both groups of humpback whales [20 humpback whales at the beginning of their fasting (HW–NM) and 20 humpback whales at a later stage (HW–SM)]. The table provides information on the medical conditions and affected species reported in the literature.

Genus of potential pathogen	Harboured by	Medical condition caused	Affected species	Source
*Aeromonas*	HW–NM	Pneumonia	Common bottlenose dolphin	Cusick and Bullock^50^
*Acinetobacter*	HW–SM, HW–NM	General infectious disease	Common bottlenose dolphin	Venn-Watson et al.^48^
*Corynebacterium*	HW–SM, HW–NM	General infectious disease, mortality	Common bottlenose dolphin	Venn-Watson et al.^48^
*Fusobacterium*	HW–SM, HW–NM	General infectious disease, mortality	Common bottlenose dolphin	Venn-Watson et al.^48^
*Mycoplasma*	HW–SM, HW–NM	General and respiratory infectious disease	Pinnipeds	Waltzek et al.^49^
*Pseudomonas*	HW–SM, HW–NM	Infectious respiratory disease	Common bottlenose dolphin	Venn-Watson et al.^48^
*Shewanella*	HW–NM	Infectious respiratory disease, mortality	Common bottlenose dolphin	Venn-Watson et al.^48^
*Staphylococcus*	HW–SM, HW–NM	Pneumonia	Common bottlenose dolphin	Venn-Watson et al.^47,48^
*Stenotrophomonas*	HW–SM, HW–NM	Infectious respiratory disease, mortality	Common bottlenose dolphin	Venn-Watson et al.^48^
*Streptococcus*	HW–SM, HW–NM	Infectious respiratory disease	Common bottlenose dolphin	Venn-Watson et al.^48^

## Discussion

We showed that the microbiota of whale blow samples were significantly different to those of their surrounding seawater (Fig. 2, Table 2). While some similarities between communities exist, these findings confirm previous reports by Bik et al.^1^^,^ Apprill et al.^2^ and Raverty et al.^4^^,^ who also found the bacterial community composition of cetacean blow to be different to seawater. Moreover, we conclude that the bacterial communities of blow samples of HW at a later stage of their fasting were significantly altered compared with those at the beginning of their migration (Figs. 2, 3, 4). Different techniques in sample collection, DNA extraction, PCR cycling as well as two separate sequencing runs of the two groups of samples might have contributed to the differences seen in the bacterial composition of the blow. We applied appropriate statistical techniques (e.g. subsampling, PIT-trap resampling) to minimise the impact of these factors on the alpha- and beta-diversity measurement determined here. In addition, the bacterial sequences of the seawater samples taken from the two sampling sites, but processed in the different manners outlined above, were similar in their alpha diversity indicating that sample processing and sequencing runs might overall have a negligible impact for our study. We therefore provide an indication that the HW at the beginning of their fasting exhibited a significantly higher richness and diversity in their blow microbiota (Table 1) than those from a later stage of fasting.

We postulate that the relatively rich and diverse whale blow microbiota at the beginning of their fasting reflected an uncompromised, and therefore stable physiological state of the airways. The whales left their feeding grounds in Antarctica only a few weeks before the sample collection of Pirotta et al.^3^ and Owen et al.^51^ presented evidence of feeding activity of HW off the south coast of New South Wales. Consequently, the whales had likely just entered a fasting state and hence were in their “nutritional prime”. In contrast, the comparably low bacterial diversity, richness and number of core taxa of whales later in their migration is correlated to their prolonged fasting. As HW lose 25–50% of their body weight during their annual migration^34,52,53^, and are therefore susceptible to exhaustion of their energy stores before they resume feeding^54^^,^ the whales’ physiological state and hence their immune system may be compromised. This state may cause a change in the composition of their airway microbiota. Studies on mice and humans showed that the airways did not need to be acutely infected to show changes in their microbial composition^13,14^. Even in healthy or subclinically infected individuals, a shift in immunological parameters resulted in a change of the diversity of microbiota.

The loss of energy stores, metabolic demands on the host and impacts on physiology and immune status due to prolonged fasting may have become increasingly relevant in recent decades. While the population of East Australian HW is growing by almost 11% per year and supposedly approaching their carrying capacity^55^^,^ the population density of the whales’ main prey, Antarctic krill, are decreasing. Atkinson et al.^56^ estimated a 70% decrease in krill numbers since the 1970s. Growing whale numbers together with shrinking prey density could potentially exacerbate the whales’ efforts to renew their energy stores in the Antarctic feeding grounds, leaving them more vulnerable to the consequences of fasting-related exhaustion in the future.

The number and abundance of core zOTUs and genera was higher in the blow of whales at the beginning compared to the later stage of their fasting (Tables 3, 4). This observation is correlated to the reduced diversity and richness of the whales in the late fasting stage. Apprill et al.^2^ did an analysis of core taxa in the blow of Northern hemisphere HW and found 25 core maximum entropy distribution (MED) sequences in 100% of samples. While they did not look at 80% core microbiota thresholds and their MED sequences are not directly comparable to the zOTUs produced in this study, Apprill et al.^2^ likely sampled non-fasting individuals. They performed sample collection in the North Atlantic north of Cape Cod, Massachusetts, USA, in July, and in the North Pacific near Vancouver Island, British Columbia, Canada, in August and off the coast of Washington State, USA, in September. These regions are part of the high-latitude summer feeding areas of the HW that reach up to the Arctic^57^. In autumn, the whales start making their way to tropical waters near the Caribbean^57^. Consequently, sample collection by Apprill et al.^2^ took place in the feeding grounds of the whales right before the beginning of the migration to their breeding grounds and when energy stores were expected to have filled up.

We found 13 zOTUs in the blow of at least 80% of whales at the beginning of their fasting that were associated with seawater. A similar finding was described in Apprill et al.^2^ where three out of 25 MED sequences were related to seawater. It is possible that these zOTUs were mixed in with the blow when the whale exhaled and thus were contaminants. However, according to Apprill et al.^2^ seawater regularly enters the airways of whales between breaths and thus may represent ‘seawater lavages of the upper respiratory tract seeded with condensed exhalation‘. Therefore, these seawater-related zOTUs may also be true inhabitants of the airways, either of transient or more permanent nature. However, as we were not able to distinguish between contamination and ‘seawater lavage’ at this point, we decided not to include these 13 zOTUs into our conservative estimate of the core.

In the blow of HW at the early and late stage of their migration, we detected several genera that include members previously reported as marine mammal pathogens (Table 5). Yet, the significance of these findings is unclear. Most bacterial genera commonly contain non-pathogenic ‘species’ alongside pathogenic ones. As 16S rRNA sequencing as used in this study rarely allows the assignment of ‘species’ identity, we are unable to estimate the true pathogenic capacity of the detected zOTUs. In addition, both groups of whales harboured a large percentage of zOTUs that belonged to unclassified genera (74% in the whales at the beginning of their migration and 55% in the whales later on). The pathogenic potential of these unclassified genera is completely unknown. Therefore, we cannot determine if one group of whales carried a larger ratio of potentially pathogenic bacteria than the other.

In conclusion our study provides some evidence that migration-associated fasting and physiological stress might correlate with a general shift and loss of diversity and richness in the airway microbiota of HW. Such a change in the bacterial community could be the direct result of a weakened immune system, which in turn may influence the rate of elimination of bacteria as well as the growth conditions within the airways enabling certain bacterial groups to proliferate. The analysis of the diversity and composition of HW blow might therefore represent a viable approach to assess the physiological state of cetaceans. In the future, the use of additional techniques to determine body condition including photogrammetry^58–60^, measuring cortisol levels^61,62^ from the whales’ blow and blubber and determining adipocyte size and number in external blubber^63^ will contribute to even better understand the correlation between blow microbiota and the physiological state of the whale.

## Methods

### Sample collection

We collected 20 blow samples of humpback whales (HumpbackSM), in Hervey Bay, Queensland, Australia, in August 2017. Sample collection and experimental procedures were approved by the University of New South Wales (UNSW Sydney) animal care and ethics committee (ACEC) (permit no. 16/81A), the Department of National Parks, Sport and Racing (under the Marine Parks Act 2004, permit no. QS2016/GS066) and the Department of Environment and Heritage Protection of Queensland (permit no. 20194/16). The experimental protocol was performed in compliance with the guidelines of the above-mentioned permits.

We collected the samples from the vessel of a commercial whale watching operator at Hervey Bay (25.2882° S, 152.7677° E), Queensland, Australia, in August 2017 (n = 20). The sampled HW’s were of unknown sex and age. Images were taken of each sampled whale to compare significant features between sampled individuals (e.g. wounds, scars and pigmentation) to ensure that each whale was only sampled once. The blow collection device followed the design of Hogg et al.^64^ and Acevedo-Whitehouse et al.^7^. For each sampling effort, six blank sterile petri dishes (14 mm diameter) were attached to the Perspex plate and a long pole. When a whale surfaced within the range of the pole near the boat, the Perspex plate was positioned about 50 cm above the blowhole of the exhaling whale to collect the droplets in the blow. Seven seawater samples were obtained by filtering 500 ml sea water collected at the blow sampling sites through a Sterivex filter unit (0.22 µm, EMD Millipore Corporation, Billerica, USA). To obtain air samples, the sampling device was exposed to air for 30 min in the absence of whale blow. The petri dishes were subsequently swabbed and then processed in the same way as the blow samples. The air samples acted as technical controls during the DNA extraction process. After sample collection the petri dishes were removed from the Perspex plate and immediately swabbed with sterile cotton swabs (Interpath Services PTY LTD, Heidelberg, Baden-Württemberg, Germany). Each cotton swab had an absorption capacity of about 70 μl of sample material and per sample one cotton swab was used in the downstream process. Face masks were worn at all times while handling the samples to avoid contaminations. The cotton swabs were stored in sterile tubes and chilled on ice until the return of the boat to the harbour. In the field lab, the shaft of the cotton swabs was trimmed and the tip transferred into a 2 ml cryovial. About 300 µl of TE-buffer (10 mM Tris–Cl pH 7.5; 1 mM EDTA) were added to each cryovial to stabilize bacterial DNA. The samples were then stored at—8 °C during transport and then at − 20 °C until processing.

### DNA extraction and 16S rRNA gene sequencing

Nucleic acids from blow samples were extracted from cotton swabs and in the case of seawater from filtering units using the FastDNA Spin Kit for Soil following the manufacturer’s protocol (MP Biomedicals, Santa Ana, California, USA). The V1-V3 region of the bacterial 16S rRNA gene was amplified using barcoded primers 27F (5′-AGAGTTTGATCMTGGCTCAG-3′) and 519R (5′-GWATTACCGCGGCKGCTG-3′)^65,66^. Each sample was amplified in duplicate and then pooled in the data analysis. The final PCR reaction volume was 25 µl consisting of 0.125 µl of each primer (40 µM, Integrated DNA Technologies, Coralville, IA, USA), 5 µl of 5X Green GoTaq Flexi Buffer (Promega, Madison, WI, USA), 0.3125 µl of Ex Taq HotStart Version (5 Units per µl, TaKaRa, Bio Inc., Shiga, Japan), 2 µl of dNTP-mix (10 mM, TaKaRa, Bio Inc., Shiga, Japan), 5 µl of MgCl_2_ (25 mM; Promega, Madison, WI, USA) and 4 µl of DNA. Negative (no DNA template) and positive controls (*Escherichia coli* genomic DNA) were included. Reactions were performed using a CFX96 TouchTM Real-Time PCR Detection System (Bio-Rad Laboratories Inc, Hercules, CA, USA) under the following program conditions: Initial denaturation at 98 °C for 2 min; then 35 cycles of denaturation at 98 °C for 10 s, annealing at 50 °C for 30 s and elongation at 72 °C for 60 s; and a final elongation at 72 °C for 5 min. PCR products were submitted to the Ramaciotti Centre for Genomics (Sydney, Australia) for purification, library preparation and paired-end amplicon sequencing (2 × 300 bp) on the Illumina MiSeq platform.

### Sequence data processing

We compared the sequences of 20 blow samples collected from HumpbackNM^3^ in May to June, 2017, with our 20 samples of HumpbackSM blow samples collected in August of the same year. The HumpbackNM sequences of Pirotta et al.^3^^,^ including controls (air), were accessed from the European Nucleotide Archive under project PRJEB23634. The seawater controls Pirotta et al.^3^ used were accessed from the NCBI Sequence Read Archive under PRJNA385736^35^.

All sequences (the HumpbackNM blow and air samples collected by Pirotta et al.^3^^,^ the SeawaterNM samples Pirotta et al.^3^ used and the HumpbackSM blow, air and seawater samples collected in this study) were analysed together. An initial quality check was performed with FastQC^67^. Paired-end reads were processed with USEARCH (version 10.240)^68^. The reads were merged and low-quality sequences (maximum number of expected errors > 2 and more than 1 ambiguous base) and those shorter than 440 bp were removed. Primers were also removed. Processed sequences of all samples were dereplicated and unique sequences were denoised and de novo clustered into zero-radius operational taxonomic units (zOTUs) with 100% similarity using the *unoise3* algorithm^69^. This is the same clustering approach utilized for amplicon sequence variants (ASV)^70^. The difference between zOTUs and ASVs does not lie in the applied similarity-threshold across sequences but in the bioinformatics program. While Callahan et al.^70^ used the program DADA2 for sequence processing and ‘species’ assemblage, we worked with USEARCH^68^.

A de novo chimera removal was included in the *unoise* step. Afterwards, remaining chimeric sequences were removed using the *uchime2* algorithm^71^ in high confidence mode with the SILVA database (version 132) as reference dataset^72^. Subsequently, processed sequences were mapped onto zOTU sequences to calculate the presence and relative abundance of each zOTU in every sample using the *otutab* command with *maxrejects* and *maxaccepts* options disabled. Representative zOTU sequences were assigned a taxonomy using the SILVA rRNA sequence database, release 132 (www.arb-silva.de)^72^ and the ribosomal database project (rdp), release 11 (https://rdp.cme.msu.edu)^73^. The taxonomical information derived from the SILVA rRNA sequence database was exclusively used to identify and delete zOTU sequences derived from mitochondria and chloroplasts.

### Data analysis

zOTUs that were identified as Archaea, chloroplasts or mitochondria were deleted from the dataset. We identified 457 zOTUs that were present in at least two air samples. As the air samples served as technical control, we considered those zOTUs as technical contaminants and deleted them from the dataset. We used the package *phyloseq* (v1.24.2) to perform a rarefaction analysis to test if a complete representation of the blow and seawater microbiota was achieved given the observed sequence sampling depths. Bacterial alpha and beta diversity of seawater and whale samples were assessed using the package *vegan* (v2.5-5) for community ecology analysis^74^. To determine alpha diversity, we calculated richness, Shannon–Wiener diversity index, Chao1 and ACE species estimator for whale and seawater samples using rarefied counts (lowest number of reads: 3432) to account for the difference in sampling depth (Table 1) and unrarefied counts (Supplementary Table S2). To visualize beta diversity, we used the Bray–Curtis dissimilarity coefficient and weighted and unweighted generalized UNIFRAC distances^75^ of unrarefied relative abundances. To determine if the composition of microbial communities of the two groups of whales and their corresponding seawater samples were significantly different from each other, a typical approach is to use PERMANOVA, the Permutational Multivariate Analysis of Variance^76^. However, the implicit assumptions made by distance-based analyses such as PERMANOVA about the mean–variance relationship of the outcome are often unrealistic in community composition data. As demonstrated by Warton et al.^77^^,^ this misspecification can lead to the confounding of location and dispersion effects, and therefore incorrect conclusions. For this reason, we instead followed the approach recommended by Warton et al.^77^ and used *mvabund* (v4.0.1)^78^ to fit negative binomial regression models to each zOTU, with the log of total sequence counts per sample included as an offset. The sum of likelihood ratio statistics was used as a community-level statistic to compare models with ‘migration’ and ‘whale blow/seawater’ as an explanatory factor to an intercept-only model. Using *mvabund*, the sum of likelihood ratio statistics and statistical significance was evaluated with anova.manyglm using pit-trap resampling^79^. Further details are provided in the supplementary materials (S3, S4). Lokmer et al.^80^ applied a similar approach for the analysis of bacterial community composition in oysters. Statistical analysis of microbial community results was performed using R statistical software (v 3.5.1) (https://cran.r-project.org/).

### Data availability

Sequence data of the blow (HumpbackSM), seawater (SeawaterSM) and the according air samples are available in the NCBI Sequence Read Archive under BioProject accession no. PRJNA521078.

## Supplementary information

Supplementary Information 1.

Supplementary Information 2.
