# Supplementary material for: Respiratory microbiota of humpback whales may be reduced in diversity and richness the longer they fast
Source: Sci Rep. 2020 Jul 28;10:12645. doi: 10.1038/s41598-020-69602-x (PMC7387350; doi:10.1038/s41598-020-69602-x)
Supplement: Supplementary file 1 — Supplementary Information 1. [file 41598_2020_69602_MOESM1_ESM.docx]

**Supplementary Information of**

**Respiratory microbiota of humpback whales may be reduced in diversity and richness the longer they fast**

Catharina Vendl^1^, Eve Slavich^1,2^, Bernd Wemheuer^5^, Tiffanie Nelson.^3^, Belinda Ferrari^4^, Torsten Thomas^5^, Tracey Rogers^1^

^1^ Evolution and Ecology Research Centre, School of Biological, Earth and Environmental Sciences, University of New South Wales, Sydney, NSW, 2052, Australia.

^2^ Stats Central, Mark Wainwright Analytical Centre, School of Mathematics and Statistics, University of New South Wales, Sydney, NSW, 2052, Australia.

^3^ Queensland Facility for Advanced Bioinformatics, Griffith University, Gold Coast, Southport, QLD, 4215, Australia.

^4^ School of Biotechnology and Biomolecular Sciences, University of New South Wales, Sydney, NSW, 2052, Australia.

^5^ Centre for Marine Bio-Innovation, School of Biological, Earth and Environmental Sciences, Sydney, NSW, 2052, Australia.

*Corresponding [c.vendl@unsw.edu.au](mailto:c.vendl@unsw.edu.au)

**Supplementary Tables**

**Table S1 Z and p-values of Kruskal-Wallis test of richness, Shannon-Wiener diversity, Chao1 and ACE species estimator of unrarefied counts.** The upper value is the Z, the lower the p-value in each cell. Those p-values with an asterisk are statistically significant.

|  | HumpbackNM | HumpbackSM | SeawaterNM |
| --- | --- | --- | --- |
| **Richness** | Z  p-value | Z  p-value | Z  p-value |
| HumpbackSM | 3.1822  0.0022* |  |  |
| SeawaterNM | -4.1703  0.0001* | -7.5536  0.0000* |  |
| SeawaterSM | -1.0372  0.1498 | -3.3287  0.0017* | 1.8431  0.0653 |
| **Diversity** |  |  |  |
| HumpbackSM | 5.2538  0.0000* |  |  |
| SeawaterNM | -0.5510  0.2908 | -6.1369  0.0000* |  |
| SeawaterSM | 1.3216  0.1863 | -2.4615  0.0277 | 1.7479  0.1207 |
| **Chao1** |  |  |  |
| HumpbackSM | 3.0107  0.0039* |  |  |
| SeawaterNM | -4.5102  0.0000* | -7.7112  0.0000* |  |
| SeawaterSM | -1.0042  0.1576 | -3.1722  0.0030* | 2.1146  0.0345 |
| **ACE** |  |  |  |
| HumpbackSM | 3.0107  0.0039* |  |  |
| SeawaterNM | -4.4194  0.0000* | -7.6204  0.0000* |  |
| SeawaterSM | -1.0648  0.1435 | -3.2327  0.0025* | 1.9887  0.0467 |

**Table S2 Number of reads and alpha diversity parameters (samples size, richness, Shannon index, Chao1 and ACE) of unrarefied counts** of samples of 20 humpback whale (HumpbackNM) and 26 seawater (SeawaterNM) samples at the beginning of their fasting and of 20 humpback whale (HumpbackSM) and 7 seawater (SeawaterSM) samples at a later stage. Numbers are after deleting putative technical contaminant zOTUs from the dataset.

| **Species** | **Sample size (n)** | **Reads per sample, mean (sd)** | **Richness (number of OTUs per sample)**  **mean (sd)** | **Shannon index (sd)** | **Chao1 species estimator (sd)** | **ACE species estimator (sd)** |
| --- | --- | --- | --- | --- | --- | --- |
| HumpbackNM | 20 | 232,201 (143,670) | 454 (228) | 5.48 (0.69) | 1027 (499) | 961 (476) |
| HumpbackSM | 20 | 21,091 (21,843) | 897 (474) | 3.33 (0.82) | 156 (187) | 177 (223) |
| SeawaterNM | 26 | 40,593 (13,628) | 1882 (347) | 5.71 (0.25) | 2269 (364) | 2043 (353) |
| SeawaterSM | 7 | 80,761 (8,648) | 1415 (62) | 5.42 (0.11) | 1541 (69) | 1465 (65) |

**Supplementary Figures**

| 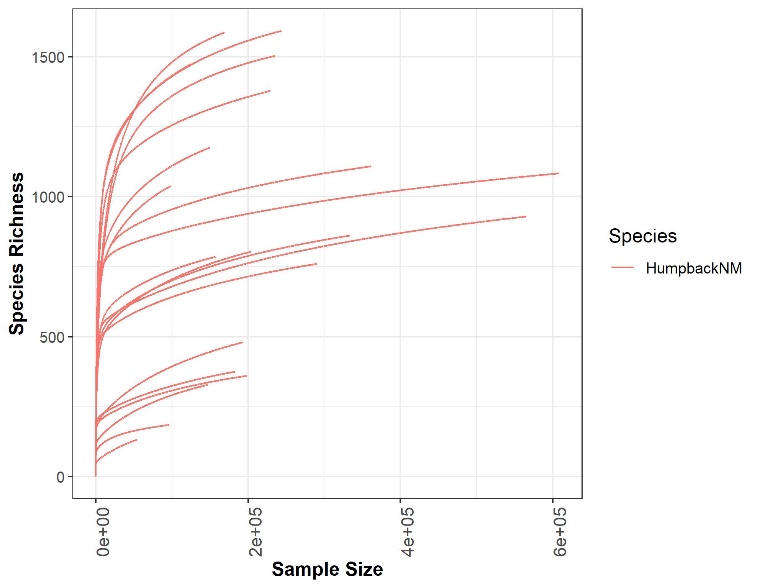 | 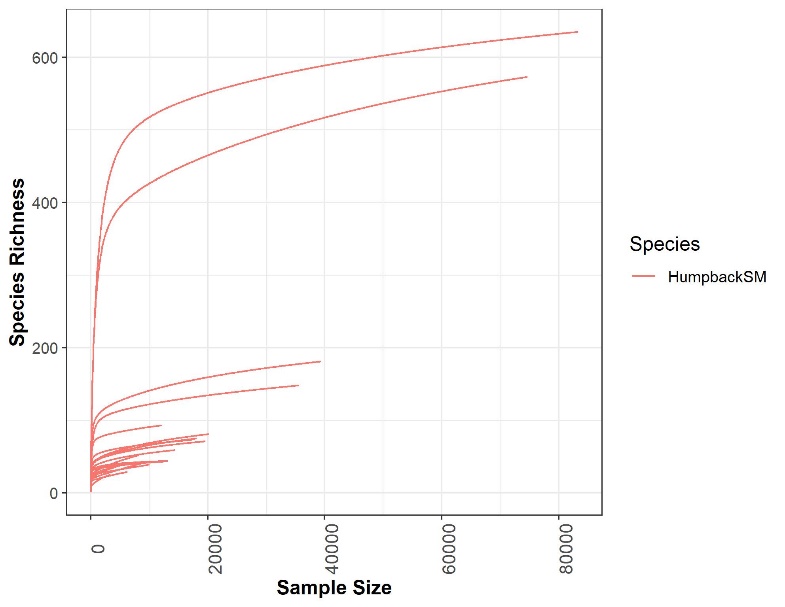 |
| --- | --- |
| 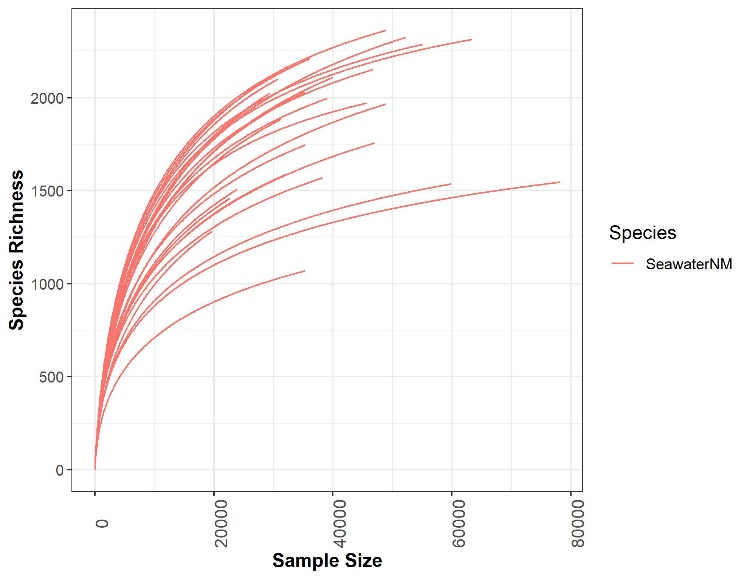 | **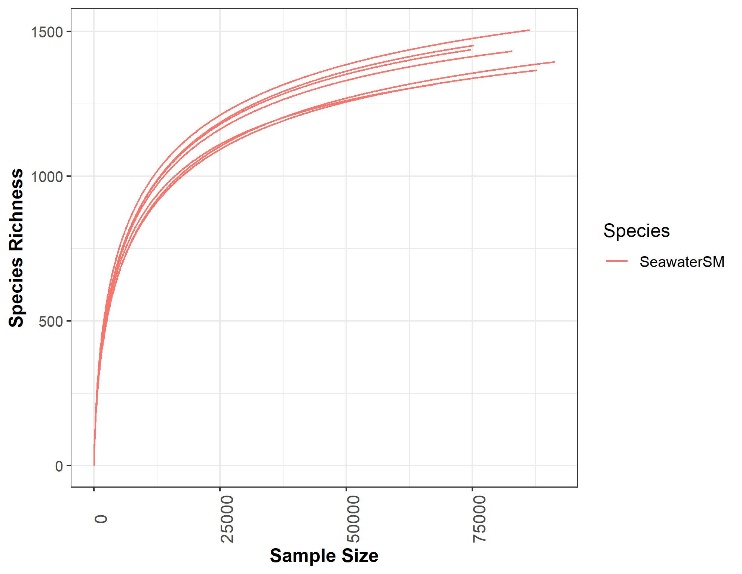** |

**FIG S1 Rarefaction curves of microbiota of the blow** of 20 humpback whale (HumpbackNM) and 26 seawater (SeawaterNM) samples at the beginning of the whales’ fasting and of 20 humpback whale (HumpbackSM) and 7 seawater (SeawaterSM) samples at a later stage after deleting putative technical contaminant zOTUs from the dataset. The microbial communities were mostly sampled to saturation.


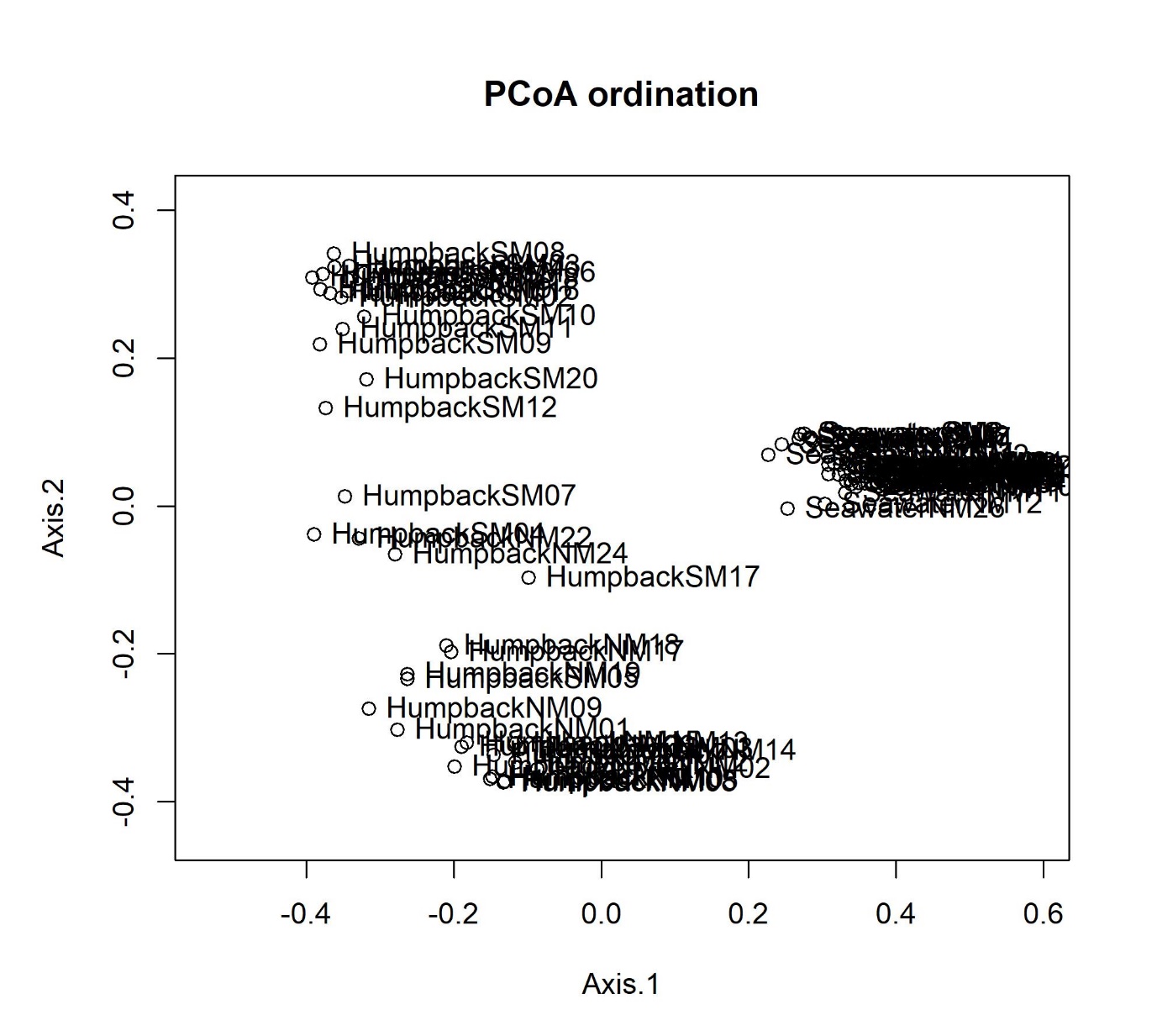
**FIG S2 PCoA plot of unweighted UNIFRAC distance of microbiota,** based on unrarefied data, found in the blow of 20 humpback whale (HumpbackNM) and 26 seawater (SeawaterNM) samples at the beginning of the whales’ fasting and of 20 humpback whale (HumpbackSM) and 7 seawater (SeawaterSM) samples at a later state after deleting putative technical contaminant zOTUs from the dataset. The seawater samples show a clear distinction to the whale blow samples, as they cluster closely together. Apart from the sample HumpbackNM24 being situated among HumpbackSM samples, the two groups of whale blow samples (HumpbackNM & HumpbackSM) are separate from each other.


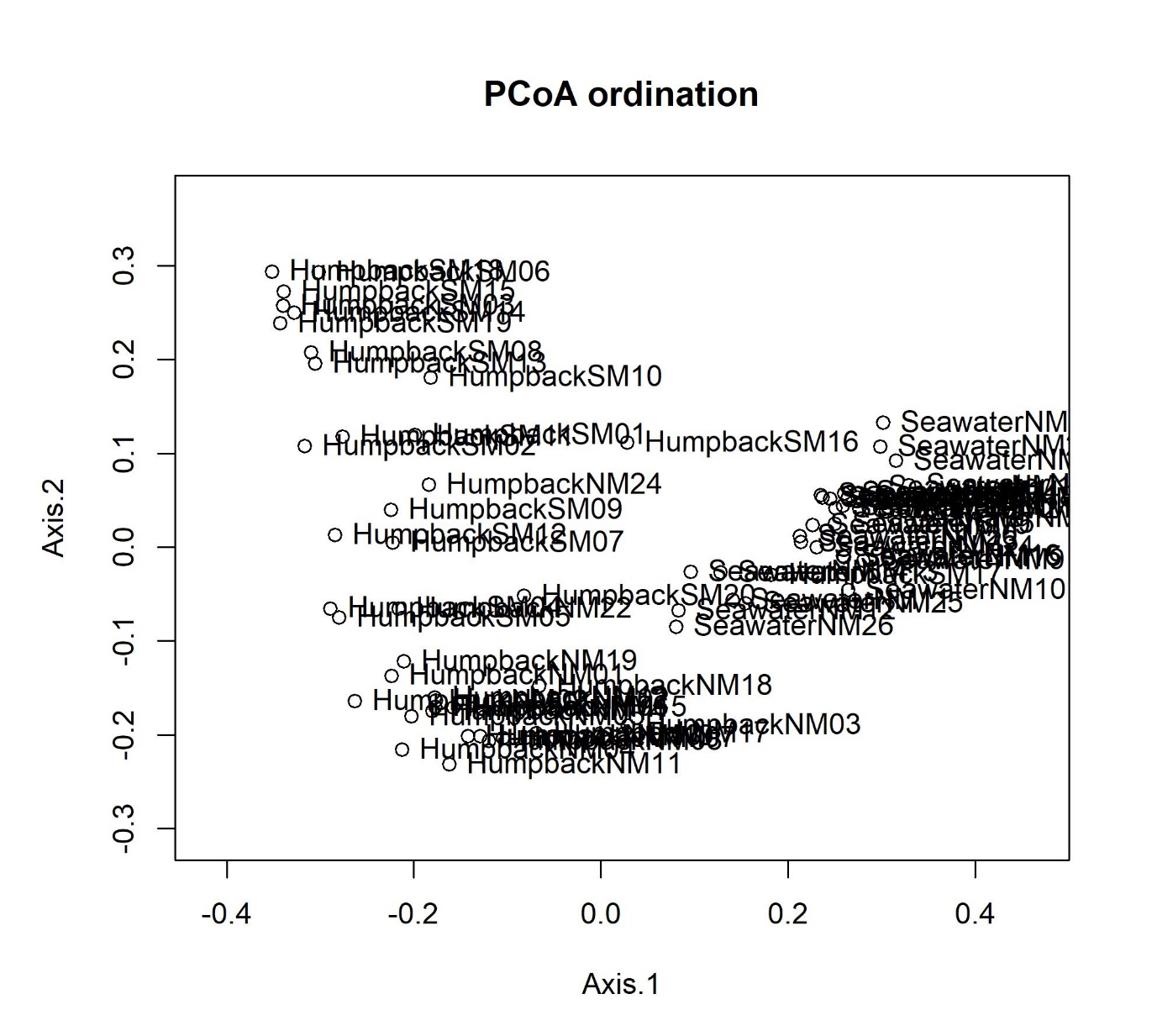
**FIG S3 PCoA plot of weighted UNIFRAC distance of microbiota,** based on unrarefied data, found in the blow of 20 humpback whale (HumpbackNM) and 26 seawater (SeawaterNM) samples at the beginning of the whales’ fasting and of 20 humpback whale (HumpbackSM) and 7 seawater (SeawaterSM) samples at a later state after deleting putative technical contaminant zOTUs from the dataset. The seawater samples show less clear clustering compared to the PCoA plot of unweighted UNIFRAC distance (FIG S1). The HumpbackSM samples are spread out but still mostly separate from HumpbackNM.


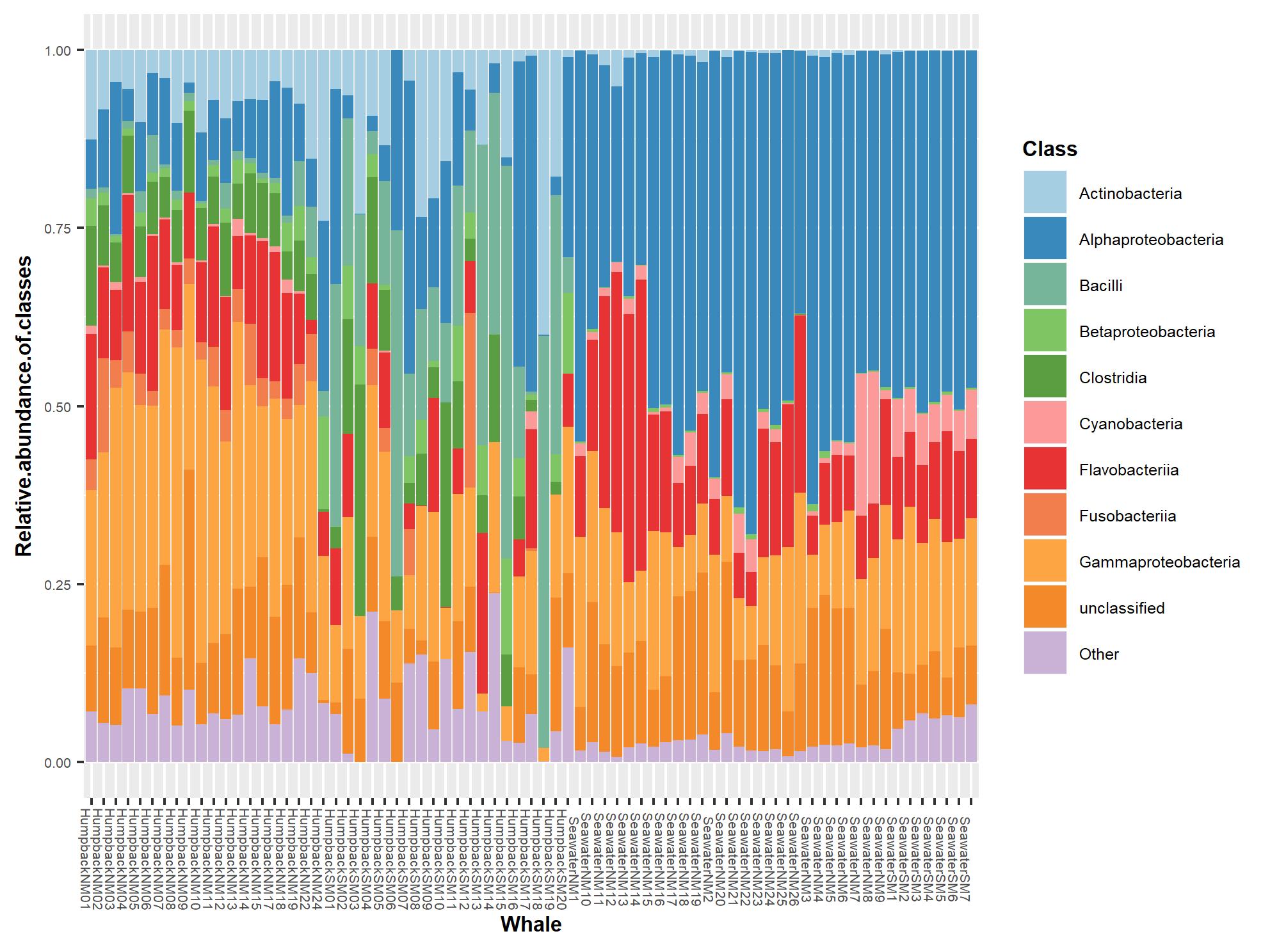


**FIG S4 Class level assignment of the microbiota detected in the blow of humpback whales** at the beginning of their fasting (HumpbackNM) and humpback whales at a later stage (HumpbackSM) and their corresponding seawater samples (SeawaterNM, SeawaterSM). ‘Other’ refers to those classes, whose mean relative abundance across samples was below 0.01%. The microbial communities of seawater showed a clear distinction in their relative abundance of classes to whale blow. HumpbackSM samples had a larger individual variability in their relative abundance of classes compared to HumpbackNM samples.

**S3 Details of statistical analysis**

Let $Y_{ij}$ be the observed count of zOTU $j$ for sample $i$. As recommended by Warton et al^1^, we modelled this outcome as a negative binomial random variable with expected value $\mathbb{E}\left( Y_{ij} \right)=\mu_{ij}$ and overdispersion parameter $\phi_{j}$, such that the variance of the outcome has a quadratic relationship with the expected value: $\text{Var}\left( Y_{ij} \right)=\mu_{ij}+\phi_{j}\mu_{ij}^{2}$.

Given that the expected count of each zOTU $\mu_{ij}$ is proportional to the total number of sequence counts for a given sample $N_{i}=\sum_{j} Y_{ij}$, we are interested in the effect of covariates on the *relative* abundance of each zOTU: $\pi_{ij}={\mu_{ij}}/{N_{i}}$. Using a log link function, we fit an intercept-only model:

$$\log\pi_{ij}=\alpha_{j}$$

and a model with a sample-specific covariate:

$$\log\pi_{ij}=\alpha_{j}+\beta_{j}x_{i},$$

separately for each $j$, where $x_{i}$ is an indicator variable for migration or whale blow / seawater as appropriate, and the adjustment for the total number of sequence counts is applied by including $\log N_{i}$ in the regression model as an offset term (since $\log\mu_{ij}=\log\pi_{ij}+\log N_{i}$). The appropriateness of the negative binomial model was confirmed by examining plots of fitted values against randomised quantile residuals^2^.

To assess the importance of the covariates, we calculated a likelihood ratio statistic for each zOTU:

$$\mathcal{L}_{j}= -2\left( {\hat{\mathcal{l}}}_{j0}-{\hat{\mathcal{l}}}_{j1} \right),$$

where ${\hat{\mathcal{l}}}_{j0}$ and ${\hat{\mathcal{l}}}_{j1}$ are the maximised values of the log-likelihood function under the intercept-only and covariate models respectively. The overall test statistic is the sum of likelihood ratio statistics across all zOTUs^1^:

$$\mathcal{L=}\sum_{j} \mathcal{L}_{j}$$

We calculated a p-value by comparing this to the distribution of such statistics calculated from 1000 datasets generated using pit-trap resampling^3^, which allows us to account for the dependence between zOTUs

1. Warton, D. I., Wright, S. T., & Wang, Y. Distance‐based multivariate analyses confound location and dispersion effects. Methods Ecol. Evol., 3, 89-101(2012).
2. Dunn, P. K., & Smyth, G. K. (1996). Randomized quantile residuals. J. Comput. Graph. Stat., 5, 236-244(1996).
3. Warton, D. I., Thibaut, L. & Wang, Y. A. The pit-trap - a "model-free" bootstrap procedure for inference about regression models with discrete, multivariate responses. PLoS One 12, e0181790 (2017).
