# Supplementary material for: Respiratory microbiota of humpback whales may be reduced in diversity and richness the longer they fast
Source: Sci Rep. 2020 Jul 28;10:12645. doi: 10.1038/s41598-020-69602-x (PMC7387350; doi:10.1038/s41598-020-69602-x)
Supplement: Supplementary file 2 — Supplementary Information 2. [file 41598_2020_69602_MOESM2_ESM.docx]

**Supplementary Information (Supplementary_S4_RCode_Analysis_Whale.blow.migration) of**

**Respiratory microbiota of humpback whales may be reduced in diversity and richness the longer they fast**

Catharina Vendl^1^, Eve Slavich^1,2^, Bernd Wemheuer^5^, Tiffanie Nelson.^3^, Belinda Ferrari^4^, Torsten Thomas^5^, Tracey Rogers^1^

^1^ Evolution and Ecology Research Centre, School of Biological, Earth and Environmental Sciences, University of New South Wales, Sydney, NSW, 2052, Australia.

^2^ Stats Central, Mark Wainwright Analytical Centre, School of Mathematics and Statistics, University of New South Wales, Sydney, NSW, 2052, Australia.

^3^ Queensland Facility for Advanced Bioinformatics, Griffith University, Gold Coast, Southport, QLD, 4215, Australia.

^4^ School of Biotechnology and Biomolecular Sciences, University of New South Wales, Sydney, NSW, 2052, Australia.

^5^ Centre for Marine Bio-Innovation, School of Biological, Earth and Environmental Sciences, Sydney, NSW, 2052, Australia.

*Corresponding [c.vendl@unsw.edu.au](mailto:c.vendl@unsw.edu.au)

**# Title: "Supplementary_S4_RCode_Analysis_Whale.blow.migration"**

**# Authors: "Eve Slavich and Catharina Vendl"**

####################

############Load libraries

options(install.packages.check.source = "no")

source('http://bioconductor.org/biocLite.R')

biocLite('phyloseq')

install.packages("stringi",type="win.binary")

install.packages("stringi", repos="http://cran.rstudio.com/", dependencies=TRUE)

library(devtools)

install_github("microbiome/microbiome")

library(BiocInstaller)

source("http://www.bioconductor.org/biocLite.R")

useDevel()

biocLite("microbiome")

library(pracma)

library(fossil)

library(indicspecies)

library(RColorBrewer)

library(microbiome)

library(hablar)

library(digest)

library(MASS)

library(mvabund)

library(reshape2)

library(dplyr)

library(ggplot2)

library(GGally)

library(vegan)

library(tidyr)

library(pillar)

library(tibble)

#library(multcomp)

library(phyloseq)

library(data.table)

library(stringi)

library(fansi)

library(RAM)

library(dunn.test)

library(pheatmap)

library(seqinr)

##########################################

### Data Reading

full.dat.migration = read.csv("AllSamples_unoise_otu_table_Silva_2.csv",header=T, sep=',')

###Filter out zOTU that are chloroplasts

full.dat.migration.2 = full.dat.migration %>% filter(!Order=='Chloroplast')

full.dat.migration.3 = full.dat.migration.2 %>% filter(!Family=='Mitochondria')

#Split Silva-tax from full.dat.migration

attributes.migration_Silva = full.dat.migration.3[,c(1,128:134)]

attributes.migration_Silva.2 = sapply(attributes.migration_Silva, as.character)

#Delete tax of full.dat.migration

dat.migration = full.dat.migration.3[,-c(128:134)]

#Read in rdp.tax

attributes.migration_rdp = read.csv("AllSamples_unoise_nbc_rdp.csv", fill=TRUE, header=T, sep=',')

#Read in var

whaleblow_var.migration = read.csv("HWblow_var.migration.csv", header=T,sep=',')

#Turn col 'variable' into rownames

rownames(dat.migration) = dat.migration[,1]

#Create col 'sum_of_counts'

dat.migration.2 = mutate (dat.migration, sum_of_rows=rowSums(dat.migration[,2:127]))

#filter rows with 'sum_of_rows' that are smaller than 2

#(removal of OTUs without any counts and of singletons)

dat.migration.3 = dat.migration.2 %>% filter(sum_of_rows > 1)

#Determine total number of reads

sum(dat.migration.3$sum_of_rows)

#Give dat.migration.3 rownames back

rownames(dat.migration.3) = dat.migration.3$zOTU

dat.migration.4 = dat.migration.3

#Delete col 1 'variable'

dat.migration.5 = dat.migration.4[,-1]

#transpose the matrix

dat.migration_t = as.data.frame(t(dat.migration.5))

dim(dat.migration_t)

#create two new variables, one is the zOTU names (zOTU_names)

#and the other is the sample names (dat.migration_t$Sample)

zOTU_names.migration=names(dat.migration_t)

dat.migration_t$Sample = rownames(dat.migration_t)

which(colnames(dat.migration_t)=='Sample')

dat.migration_t.2 = dat.migration_t[,c(8838,1:8837)]

#merge the species/whale/replicate data to the OTU data

dat.migration_t.merge = merge(whaleblow_var.migration ,dat.migration_t.2,by="Sample")

#Reformate the data into long

dat.migration_long = melt(dat.migration_t.merge, id.migration.vars=c("Sample","Species","Whale","Replicate"))

#Sum the replicates on the same whale and control

dat.migration_counts = dat.migration_long %>%

group_by(Species,Whale,variable) %>%

summarise(sum_of_counts = sum(value))

dat.migration_counts.2 = dat.migration_counts %>%filter(!variable=='Replicate')

#Reformat back to wide

migration = dcast(dat.migration_counts.2, Species+Whale~variable,value.var.migration="sum_of_counts")

#Which OTUs can be found in which whale species?

OTU_found_in_species.migration = dat.migration_counts.2 %>%

group_by (Species, variable) %>%

summarise(Total_OTU_per_species.migration = sum(sum_of_counts)) %>%

mutate(OTU_found_in_species.migration = Total_OTU_per_species.migration>0)

#Turn into long format again

OTU_found_in_species.migration.2 = dcast (OTU_found_in_species.migration,variable~Species ,

value.var.migration = "OTU_found_in_species.migration")

#paste0 concatenate ('verkettet') vectors after converting to character, creates column names: e.g. 'OTU_found_inAir' etc.

names(OTU_found_in_species.migration.2)[2:7] = paste0("OTU_found_in",names(OTU_found_in_species.migration.2)[2:7])

#'merge' merges two dataframes 'by' common column or row name

dat.migration.3 = merge(dat.migration_counts.2, OTU_found_in_species.migration.2 , type="left", by = "variable")

#Reformat back to wide

dat.migration_counts_Wide.2 = dcast(dat.migration_counts.2, Species+Whale~variable,value.var="sum_of_counts")

#For calculating relative abundance

rownames(dat.migration_counts_Wide.2) = dat.migration_counts_Wide.2$Whale

dat.migration_counts_Wide.3=dat.migration_counts_Wide.2 [,-c(1:2)]

#Calculate relative abundance

dat.migration_counts_Wide.3.rel.abund = dat.migration_counts_Wide.3/rowSums(dat.migration_counts_Wide.3)

##log transform

dat.migration_counts_Wide.3.rel.abund.log=log(dat.migration_counts_Wide.3.rel.abund+1)

############################################################

###Filering of air contaminants

dat.migration_counts_Wide_6 = dat.migration_counts_Wide_5[,-8502]

###Seperate rows of Air

###AirSM

dat.migration_counts_AirSM = dat.migration_counts_Wide_6[c(7:12),]

#Transpose

dat.migration_counts_AirSM_t = as.data.frame(t(dat.migration_counts_AirSM))

#View(dat.migration_counts_AirSM_t)

#Turn rownames into col

dat.migration_counts_AirSM_t$variable = rownames(dat.migration_counts_AirSM_t)

#Create sum of rows

dat.migration_counts_AirSM_t = dat.migration_counts_AirSM_t %>% mutate (sum_of_rows = rowSums(dat.migration_counts_AirSM_t[,1:6]))

dat.migration_counts_AirSM_t.no0 = dat.migration_counts_AirSM_t %>% filter (!sum_of_rows == 0)

OTUs.AirSM.100 = dat.migration_counts_AirSM_t.no0$variable

###Check how common OTUs.AirSM.100 are in HWSM

dat.migration_counts_Wide_HWSM.AirSM = dat.migration_counts_Wide_6[c(7:12,33:52),]

dat.migration_counts_Wide_HWSM.AirSM_2 = dat.migration_counts_Wide_HWSM.AirSM[,OTUs.AirSM.100]

dat.migration_counts_Wide_HWSM.AirSM_2.rel.abund = dat.migration_counts_Wide_HWSM.AirSM_2/rowSums(dat.migration_counts_Wide_HWSM.AirSM_2)

####################################################################################

###AirNM

dat.migration_counts_AirNM = dat.migration_counts_Wide_6[c(1:6),]

#Transpose

dat.migration_counts_AirNM_t = as.data.frame(t(dat.migration_counts_AirNM))

#Turn rownames into col

dat.migration_counts_AirNM_t$variable = rownames(dat.migration_counts_AirNM_t)

#Create sum of rows

dat.migration_counts_AirNM_t = dat.migration_counts_AirNM_t %>% mutate (sum_of_rows = rowSums(dat.migration_counts_AirNM_t[,1:6]))

dat.migration_counts_AirNM_t.no0 = dat.migration_counts_AirNM_t %>% filter (!sum_of_rows == 0)

dim(dat.migration_counts_AirNM_t.no0)

OTUs.AirNM.100 = dat.migration_counts_AirNM_t.no0$variable

###Check how common OTUs.AirNM.100 are in HWNM

dat.migration_counts_Wide_HWNM.AirNM = dat.migration_counts_Wide_6[c(1:6,13:32),]

dat.migration_counts_Wide_HWNM.AirNM_2 = dat.migration_counts_Wide_HWNM.AirNM[,OTUs.AirNM.100]

dat.migration_counts_Wide_HWNM.AirNM_2.rel.abund = dat.migration_counts_Wide_HWNM.AirNM_2/rowSums(dat.migration_counts_Wide_HWNM.AirNM_2)

OTUs.AirNM.101 = setdiff(OTUs.AirNM.100, OTUs.keep.HWNM.SWNM)

intersect(OTUs.AirNM.100, OTUs.AirSM.100)

#Does OTUs.AirNM.100 appear a lot in dat.migration_counts_Wide_HWSM.SW.AirSM_2.rel.abund?

dat.migration_counts_Wide_HWSM.AirSM.2 = dat.migration_counts_Wide_HWSM.AirSM[,OTUs.AirNM.101]

#Does OTUs.AirSM.100 appear a lot in dat.migration_counts_Wide_HWNM.SW.AirNM_2.rel.abund?

dat.migration_counts_Wide_HWNM.AirNM.2 = dat.migration_counts_Wide_HWNM.AirNM[,OTUs.AirSM.101]

##########################################

###Remove OTUs.AirSM.101 from dat.migration_counts_Wide_HWSM.SW.AirSM

###Delete OTUs.AirSM.101 from dat.migration_counts_Wide_HWSM.SW.AirSM

OTUs.HWSM.SWSM.100 = names(dat.migration_counts_Wide_HWSM.SW.AirSM)

OTUs.HWSM.SWSM.101 = setdiff(OTUs.HWSM.SWSM.100, OTUs.AirSM.101)

dat.migration_counts_Wide_HWSM.SWSM_Air.fil = dat.migration_counts_Wide_HWSM.SW.AirSM[,OTUs.HWSM.SWSM.101]

#####################################

#Now reconstruct original dataframe.SM with contaminant zOTUs as 0

conts.SM.Air = data.frame(matrix(0, ncol = 145, nrow = 33))

names(conts.SM.Air) = OTUs.AirSM.101

rownames.HWSM.SWSM_Air.fil = rownames(dat.migration_counts_Wide_HWSM.SWSM_Air.fil)

dat.migration.fil.HWSM.Air.fil = bind_cols(dat.migration_counts_Wide_HWSM.SWSM_Air.fil,conts.SM.Air)

dat.migration.fil.HWSM.Air.fil$Whale = rownames.HWSM.SWSM_Air.fil

##########################################

##########################################

###Remove OTUs.AirNM.101 from dat.migration_counts_Wide_HWNM.SW.AirNM

###Delete OTUs.AirNM.101 from dat.migration_counts_Wide_HWNM.SW.AirNM

OTUs.HWNM.SWNM.100 = names(dat.migration_counts_Wide_HWNM.SW.AirNM)

OTUs.HWNM.SWNM.101 = setdiff(OTUs.HWNM.SWNM.100, OTUs.AirNM.101)

dat.migration_counts_Wide_HWNM.SWNM_Air.fil = dat.migration_counts_Wide_HWNM.SW.AirNM[,OTUs.HWNM.SWNM.101]

#####################################

#Now reconstruct original dataframe.NM with contaminant zOTUs as 0

conts.NM.Air = data.frame(matrix(0, ncol = 400, nrow = 52))

names(conts.NM.Air) = OTUs.AirNM.101

rownames.HWNM.SWNM_Air.fil = rownames(dat.migration_counts_Wide_HWNM.SWNM_Air.fil)

dat.migration.fil.HWNM.Air.fil = bind_cols(dat.migration_counts_Wide_HWNM.SWNM_Air.fil,conts.NM.Air)

dat.migration.fil.HWNM.Air.fil$Whale = rownames.HWNM.SWNM_Air.fil

###########################################

###Combine dat.migration.fil.HWNM.Air.fil and dat.migration.fil.HWSM.Air.fil

dat.migration.Air.fil = bind_rows(dat.migration.fil.HWNM.Air.fil,dat.migration.fil.HWSM.Air.fil)

rownames(dat.migration.Air.fil) = dat.migration.Air.fil$Whale

dat.migration.Air.fil.2 = dat.migration.Air.fil[,-8502]

dat.migration.Air.fil.4 = dat.migration.Air.fil.2[, colSums(dat.migration.Air.fil.2 != 0) > 0]

#######################################################################################

###Alpha diversity after Air filtering!

rowSums(dat.migration.Air.fil.4)

mean(rowSums(dat.migration.Air.fil.4))

sd(rowSums(dat.migration.Air.fil.4))

min(rowSums(dat.migration.Air.fil.4[,1:8037]))

dat.migration.Air.fil.4$Whale = rownames(dat.migration.Air.fil.4)

###Prepare whaleblow_var.migration.sh for use

#Delete col 1 and 4 from whaleblow_var.migration.sh

whaleblow_var.migration.sh_2 = whaleblow_var.migration.sh [,-c(1,4)]

#Remove air sample rows

whaleblow_var.migration.sh_Air.fil = whaleblow_var.migration.sh_2 %>% filter (!Species=='AirNM')

whaleblow_var.migration.sh_Air.fil_2 = whaleblow_var.migration.sh_Air.fil %>% filter (!Species=='AirSM')

#Delete HumpbackNM16

whaleblow_var.migration.sh_Air.fil_3 = whaleblow_var.migration.sh_Air.fil_2 %>% filter (!Whale=='HumpbackNM16')

#Join with whaleblow_var.migration.sh.fil

dat.migration.Air.fil.4_2 = whaleblow_var.migration.sh_Air.fil_3 %>%

left_join(dat.migration.Air.fil.4, by='Whale')

#Create sum_of_rows

dat.migration.Air.fil.4_3 = dat.migration.Air.fil.4_2 %>%

mutate(sum_of_counts=rowSums(dat.migration.Air.fil.4_2[,3:8039]))

Overview.sumofcounts.Air.fil = dat.migration.Air.fil.4_3 %>% group_by(Species) %>%

summarize(min(sum_of_counts),

max(sum_of_counts),

mean(sum_of_counts),

sd(sum_of_counts))

############

###Alpha diversity with rarefied counts

#Delete col 'sum_of_counts'

dat.migration_counts.Air.fil.alpha = dat.migration.Air.fil.4_3[,-8040]

###Turn col 'Whale' into rownames

rownames(dat.migration_counts.Air.fil.alpha) = dat.migration_counts.Air.fil.alpha[,2]

dat.migration_counts.Air.fil.alpha.2 = dat.migration_counts.Air.fil.alpha[,-c(1,2)]

# Rarefaction

min(rowSums(dat.migration_counts.Air.fil.alpha.2))

dat.migration_counts.Air.fil.alpha.rff = rrarefy(x = dat.migration_counts.Air.fil.alpha.2, sample = 3432)

rowSums(dat.migration_counts.Air.fil.alpha.rff)

# Calculating alpha diversity

species_richness.Air.fil = NULL

species_diversity.Air.fil = NULL

species_estimator.Air.fil = NULL

for(i in 1:100)

{

print(i)

dat.migration_counts.Air.fil.alpha.rff = rrarefy(x = dat.migration_counts.Air.fil.alpha.2, sample = 3432)

species_richness.Air.fil = rbind(species_richness.Air.fil, specnumber(dat.migration_counts.Air.fil.alpha.rff))

species_diversity.Air.fil = rbind(species_diversity.Air.fil, diversity(dat.migration_counts.Air.fil.alpha.rff))

species_estimator.Air.fil = rbind(species_estimator.Air.fil, estimateR(dat.migration_counts.Air.fil.alpha.rff)[2,])

}

species_richness.Air.fil_avg = colMeans(species_richness.Air.fil)

species_diversity.Air.fil_avg = log(colMeans(exp(species_diversity.Air.fil)))

species_estimator.Air.fil_avg = colMeans(species_estimator.Air.fil)

alpha.migration.Air.fil = data.frame(species_richness.Air.fil_avg, species_diversity.Air.fil_avg, species_estimator.Air.fil_avg)

#Test diversity and richness

richness.migration.Air.fil = alpha.migration.Air.fil

richness.migration.Air.fil$Whale = rownames(richness.migration.Air.fil)

dim(richness.migration.Air.fil)

#73 4

Species.Air.fil = c(rep('HumpbackSM',20), rep('SeawaterSM',7),rep('SeawaterNM',26),rep('HumpbackNM',20))

richness.migration.Air.fil.2 = cbind(Species.Air.fil, richness.migration.Air.fil)

#mean richness.HWSM of migration

mean(richness.migration.Air.fil.2[1:20,2])

sd(richness.migration.Air.fil.2[1:20,2])

#mean richness.SeawaterSM of migration

mean(richness.migration.Air.fil.2[21:27,2])

sd(richness.migration.Air.fil.2[21:27,2])

#mean richness.SeawaterNM

mean(richness.migration.Air.fil.2[28:53,2])

sd(richness.migration.Air.fil.2[28:53,2])

#mean richness.HWNM of migration

mean(richness.migration.Air.fil.2[54:73,2])

sd(richness.migration.Air.fil.2[54:73,2])

##########Check richness

#Dunn test

dunn.test(richness.migration.Air.fil.2$species_richness.Air.fil_avg, richness.migration.Air.fil.2$Species.Air.fil, method='holm')

##########Check Shannon Wiener Diversity

#mean richness.HWNM of migration

mean(richness.migration.Air.fil.2[54:73,3])

sd(richness.migration.Air.fil.2[54:73,3])

#mean richness.HWSM of migration

mean(richness.migration.Air.fil.2[1:20,3])

sd(richness.migration.Air.fil.2[1:20,3])

#mean richness.SeawaterNM

mean(richness.migration.Air.fil.2[28:53,3])

sd(richness.migration.Air.fil.2[28:53,3])

#mean richness.SeawaterSM of migration

mean(richness.migration.Air.fil.2[21:27,3])

sd(richness.migration.Air.fil.2[21:27,3])

#Dunn test

dunn.test(richness.migration.Air.fil.2$species_diversity.Air.fil_avg, richness.migration.Air.fil.2$Species.Air.fil, method='holm')

###Chao1

#Calculate Chao1 on rarefied data

#Chao species estimator for abundance

Chao1.Air.fil = apply(dat.migration_counts.Air.fil.alpha.rff, 1, chao1)

Chao1.Air.fil.df = data.frame(Species.Air.fil,Chao1.Air.fil)

#Mean chao1

Chao1.Air.fil.mean = aggregate(Chao1.Air.fil.df[2], list(Chao1.Air.fil.df$Species.Air.fil), mean)

#sd Chao1

Chao1.Air.fil.sd = aggregate(Chao1.Air.fil.df[2], list(Chao1.Air.fil.df$Species.Air.fil), sd)

#Sign. difference?

dunn.test(Chao1.Air.fil.df$Chao1.Air.fil, Chao1.Air.fil.df$Species.Air.fil, method='holm')

###ACE

#Calculate ACE on rarefied data

#ACE species estimator for abundance

ACE.Air.fil = apply(dat.migration_counts.Air.fil.alpha.rff, 1, ACE)

ACE.Air.fil.df = data.frame(Species.Air.fil,ACE.Air.fil)

#Mean ACE

ACE.Air.fil.mean = aggregate(ACE.Air.fil.df[2], list(ACE.Air.fil.df$Species.Air.fil), mean)

#sd ACE

ACE.Air.fil.sd = aggregate(ACE.Air.fil.df[2], list(ACE.Air.fil.df$Species.Air.fil), sd)

#Sign. difference?

dunn.test(ACE.Air.fil.df$ACE.Air.fil, ACE.Air.fil.df$Species.Air.fil, method='holm')

############

############

###Alpha diversity without rarefying

###Richness

Richness.unrare = as.data.frame(specnumber(dat.migration_counts.Air.fil.alpha.2))

names(Richness.unrare) = 'Richness'

#mean richness.HWSM of migration

mean(Richness.unrare[1:20,])

sd(Richness.unrare[1:20,])

#mean richness.SeawaterSM of migration

mean(Richness.unrare[21:27,])

sd(Richness.unrare[21:27,])

#mean richness.SeawaterNM

mean(Richness.unrare[28:53,])

sd(Richness.unrare[28:53,])

#mean richness.HWNM of migration

mean(Richness.unrare[54:73,])

sd(Richness.unrare[54:73,])

##########Check richness

#Dunn test

dunn.test(Richness.unrare$Richness, richness.migration.Air.fil.2$Species.Air.fil, method='holm')

##########Check Shannon Wiener Diversity

###Diversity

Diversity.unrare = as.data.frame(diversity(dat.migration_counts.Air.fil.alpha.2))

names(Diversity.unrare) = 'Diversity'

#mean richness.HWNM of migration

mean(Diversity.unrare[54:73,])

sd(Diversity.unrare[54:73,])

#mean richness.HWSM of migration

mean(Diversity.unrare[1:20,])

sd(Diversity.unrare[1:20,])

#mean richness.SeawaterNM

mean(Diversity.unrare[28:53,])

sd(Diversity.unrare[28:53,])

#mean richness.SeawaterSM of migration

mean(Diversity.unrare[21:27,])

sd(Diversity.unrare[21:27,])

#Dunn test

dunn.test(Diversity.unrare$Diversity, richness.migration.Air.fil.2$Species.Air.fil, method='holm')

##########Check Simpson's index of diversity

###Diversity

Diversity.simps.unrare = as.data.frame(diversity(dat.migration_counts.Air.fil.alpha.2, index='simpson'))

names(Diversity.simps.unrare) = 'Diversity.simps'

#mean richness.HWNM of migration

mean(Diversity.simps.unrare[54:73,])

sd(Diversity.simps.unrare[54:73,])

#mean richness.HWSM of migration

mean(Diversity.simps.unrare[1:20,])

sd(Diversity.simps.unrare[1:20,])

#mean richness.SeawaterNM

mean(Diversity.simps.unrare[28:53,])

sd(Diversity.simps.unrare[28:53,])

#mean richness.SeawaterSM of migration

mean(Diversity.simps.unrare[21:27,])

sd(Diversity.simps.unrare[21:27,])

#Dunn test

dunn.test(Diversity.simps.unrare$Diversity.simps, richness.migration.Air.fil.2$Species.Air.fil, method='holm')

###Chao1

#Calculate Chao1 on rarefied data

#Chao species estimator for abundance

Chao1.Air.fil.unrare = apply(dat.migration_counts.Air.fil.alpha.2, 1, chao1)

Chao1.Air.fil.unrare.df = data.frame(Species.Air.fil,Chao1.Air.fil.unrare)

#Mean chao1

Chao1.Air.fil.unrare.mean = aggregate(Chao1.Air.fil.unrare.df[2], list(Chao1.Air.fil.unrare.df$Species.Air.fil), mean)

#sd Chao1

Chao1.Air.fil.unrare.sd = aggregate(Chao1.Air.fil.unrare.df[2], list(Chao1.Air.fil.unrare.df$Species.Air.fil), sd)

#Sign. difference?

dunn.test(Chao1.Air.fil.unrare.df$Chao1.Air.fil.unrare, Chao1.Air.fil.unrare.df$Species.Air.fil, method='holm')

###ACE

#Calculate ACE on rarefied data

#ACE species estimator for abundance

ACE.Air.unrare.fil = apply(dat.migration_counts.Air.fil.alpha.2, 1, ACE)

ACE.Air.fil.unrare.df = data.frame(Species.Air.fil,ACE.Air.unrare.fil)

#Mean ACE

ACE.Air.fil.unrare.mean = aggregate(ACE.Air.fil.unrare.df[2], list(ACE.Air.fil.df$Species.Air.fil), mean)

#sd ACE

ACE.Air.fil.unrare.sd = aggregate(ACE.Air.fil.unrare.df[2], list(ACE.Air.fil.df$Species.Air.fil), sd)

#Sign. difference?

dunn.test(ACE.Air.fil.unrare.df$ACE.Air.unrare.fil, ACE.Air.fil.unrare.df$Species.Air.fil, method='holm')

################################################

#######phyloseq (Create rarefaction curves)

#dat.migration.Air.fil.4 is air.filtered count dataset

#whaleblow_var.migration.sh_Air.fil_3

OTUs.migration.Air.fil = names(dat.migration.Air.fil.4[,1:8037])

###Filter contaminant OTUs from dim(attributes.migration_Silva.2) and delete OTU species

rownames(attributes.migration_Silva.2) = attributes.migration_Silva.2[,1]

attributes.migration_Silva.3 = as.data.frame(attributes.migration_Silva.2[,-c(1,8)])

attributes.migration_Silva.4 = attributes.migration_Silva.3[OTUs.migration.Air.fil,]

dim(attributes.migration_Silva.4)

#Delete col 'Whale

dim(dat.migration.Air.fil.4)

dat.migration.Air.fil.4_12 = dat.migration.Air.fil.4[,-8038]

#Transpose dat.migration.counts.fil.11

dat.migration.Air.fil.4_12_t = as.data.frame(t(dat.migration.Air.fil.4_12))

dim(dat.migration.Air.fil.4_12_t)

## check what class/type the files are

class(dat.migration.Air.fil.4_12_t)

class(attributes.migration_Silva.4)

rownames(whaleblow_var.migration.sh_Air.fil_3) = whaleblow_var.migration.sh_Air.fil_3$Whale

## they need to become matrix files to work with phyloseq

whale_otu_nm = as.matrix(sapply(dat.migration.Air.fil.4_12_t, as.numeric))

whale_tax_cm = as.matrix(sapply(attributes.migration_Silva.4, as.character))

## check they are matrices

class(whale_otu_nm)

class(whale_tax_cm)

## convert files into phyloseq object types

OTU = otu_table(whale_otu_nm, taxa_are_rows = TRUE)

TAX = tax_table(whale_tax_cm)

whaledata=sample_data(data.frame(whaleblow_var.migration.sh_Air.fil_3, stringsAsFactors = F))

class(OTU)

class(TAX)

class(whaledata)

#phyloseq

sample_names(OTU)

sample_names(whaledata)

## merge the files with phyloseq

whale1 = phyloseq(OTU, TAX, whaledata)

## get an indication of what is in the file

whale1

### Look at taxonomic names of the data

length(table(tax_table(whale1)[,"Phylum"], exclude = NULL))

#30

length(table(tax_table(whale1)[,"Class"], exclude = NULL))

#63

length(table(tax_table(whale1)[,"Order"], exclude = NULL))

#162

length(table(tax_table(whale1)[,"Family"], exclude = NULL))

#295

length(table(tax_table(whale1)[,"Genus"], exclude = NULL))

#681

#'specnumber()' shows number of species per sample

whale2 <- methods::as(phyloseq::otu_table(whale1), "matrix")

if (phyloseq::taxa_are_rows(whale1)) { whale2 <- t(whale2) }

specnumber(whale2)

#########################

###Rarefaction curves

require(parallel)

ggrare <- function(physeq, step = 10, label = NULL, color = NULL, plot = TRUE, parallel = FALSE, se = TRUE) {

x <- as(otu_table(physeq), "matrix")

if (taxa_are_rows(physeq)) { x <- t(x) }

## This script is adapted from vegan `rarecurve` function

tot <- rowSums(x)

S <- rowSums(x > 0)

nr <- nrow(x)

rarefun <- function(i) {

cat(paste("rarefying sample", rownames(x)[i]), sep = "\n")

n <- seq(1, tot[i], by = step)

if (n[length(n)] != tot[i]) {

n <- c(n, tot[i])

}

y <- rarefy(x[i, ,drop = FALSE], n, se = se)

if (nrow(y) != 1) {

rownames(y) <- c(".S", ".se")

return(data.frame(t(y), Size = n, Sample = rownames(x)[i]))

} else {

return(data.frame(.S = y[1, ], Size = n, Sample = rownames(x)[i]))

}

}

if (parallel) {

out <- mclapply(seq_len(nr), rarefun, mc.preschedule = FALSE)

} else {

out <- lapply(seq_len(nr), rarefun)

}

df <- do.call(rbind, out)

## Get sample data

if (!is.null(sample_data(physeq, FALSE))) {

sdf <- as(sample_data(physeq), "data.frame")

sdf$Sample <- rownames(sdf)

data <- merge(df, sdf, by = "Sample")

labels <- data.frame(x = tot, y = S, Sample = rownames(x))

labels <- merge(labels, sdf, by = "Sample")

}

## Add, any custom-supplied plot-mapped variables

if( length(color) > 1 ){

data$color <- color

names(data)[names(data)=="color"] <- deparse(substitute(color))

color <- deparse(substitute(color))

}

if( length(label) > 1 ){

labels$label <- label

names(labels)[names(labels)=="label"] <- deparse(substitute(label))

label <- deparse(substitute(label))

}

p <- ggplot(data = data, aes_string(x = "Size", y = ".S", group = "Sample", color = color)) +

theme_bw() +

theme(plot.title = element_text(size = 14, family = "Tahoma", face = "bold"),

text = element_text(size = 12, family = "Tahoma"),

axis.title = element_text(face="bold"),

axis.text.x=element_text(size = 11)) +

scale_fill_brewer(palette = "Accent") +

theme(axis.text.x=element_text(angle=90))

p <- p + labs(x = "Sample Size", y = "Species Richness")

if (!is.null(label)) {

p <- p + geom_text(data = labels, aes_string(x = "x", y = "y", label = label, color = color),

size = 4, hjust = 0)

}

p <- p + geom_line()

if (se) { ## add standard error if available

p <- p + geom_ribbon(aes_string(ymin = ".S - .se", ymax = ".S + .se", color = NULL, fill = color), alpha = 0.2)

}

if (plot) {

plot(p)

}

invisible(p)

}

phylodiv <- function(physeq) {

## Args:

## - physeq: phyloseq class object, from which phylogeny and abundance data are extracted

x <- as(otu_table(physeq), "matrix")

if (taxa_are_rows(physeq)) { x <- t(x) }

phy <- phy_tree(physeq)

## Construct incidence matrix of the tree

incidence <- incidenceMatrix(phy)

## Order incidence matrix according to community tables

incidence <- incidence[colnames(x), ]

## Create community phylogeny matrix by multiplying (community x edge matrix)

## where cpm_{ij} gives the abundance of OTUs originating from branch j in community i.

cpm <- x %*% incidence

## Convert to incidence matrix (0/1) and multiply by edge length to obtain PD per community.

cpm[cpm > 0] <- 1

pd <- cpm %*% phy$edge.length

## Add sample data information

if (!is.null(sample_data(physeq, FALSE))) {

sdf <- as(sample_data(physeq), "data.frame")

sdf$pd <- as.vector(pd)

pd <- sdf

}

return (pd)

}

p = ggrare(whale1, step = 100, color = "Species", se = FALSE)

p = p + facet_wrap(~Species)

ggsave("rarefaction.curves.migrationAir.fil.jpg", plot = p , device = 'jpg', width = 168, height = 130, units = "mm",

dpi = 300, limitsize = TRUE)

##########################

##Get good's coverage

head(otu_table(whale1))

## need samples as rows

t.OTU.table <- t(otu_table(whale1)) # transpose the table

sample_data(whale1)

# check

#View(t.OTU.table[1:5,1:5])

dim(t.OTU.table)

#73 8,037

#install.packages("devtools")

library(devtools)

#devtools::install_github("jfq3/QsRutils")

library(QsRutils)

goods<-goods(otu_table(t.OTU.table))

goods

goods[1:5,1:3]

dim(goods)

## add on the variables to plot

var<-sample_data(whale1)

goods_var <- cbind(goods, var)

goods_var$ID <- rownames(goods_var)

goods_var[1:5,1:5]

#install_github("easyGgplot2", "kassambara")

library(easyGgplot2)

# The plot is colored by the groupName

## sort by group Sample

attach(goods_var)

goods_var = goods_var[order(Species),]

goods.coverage.Air.fil = ggplot2.scatterplot(data=goods_var, xName='Species', yName='goods', groupName="Species",

ytitle="Good's Coverage Estimator", xtitle="Species") +

theme_bw() +

theme(plot.title = element_text(size = 16, family = "Tahoma", face = "bold"),

text = element_text(size = 12, family = "Tahoma"),

axis.title = element_text(face="bold"),

axis.text.x=element_text(size = 11)) +

scale_fill_brewer(palette = "Accent")

ggsave("goods.coverage.jpg", plot = goods.coverage , device = 'jpg', width = 168, height = 150, units = "mm",

dpi = 300, limitsize = TRUE)

aggregate(goods_var[,3], list(goods_var$Species), mean)

aggregate(goods_var[,3], list(goods_var$Species), sd)

################################################

#######phyloseq

###Create 4 individual raraefaction curves for each group (HWNM, HWSM, SeawaterNM, SeawaterSM)

###Split dat.migration.Air.fil.5_t into groups

##############

#HWNM

dat.migration.Air.fil.5_t.HWNM = dat.migration.Air.fil.5_t[,1:20]

whaleblow_var.migration.sh_Air.fil_HWNM = whaleblow_var.migration.sh_Air.fil_3[c(54:73),]

## they need to become matrix files to work with phyloseq

whale_otu_nm.HWNM = as.matrix(sapply(dat.migration.Air.fil.5_t.HWNM, as.numeric))

whale_tax_cm = as.matrix(sapply(attributes.migration_Silva.4, as.character))

## check they are matrices

class(whale_otu_nm.HWNM)

class(whale_tax_cm)

## convert files into phyloseq object types

OTU.HWNM = otu_table(whale_otu_nm.HWNM, taxa_are_rows = TRUE)

TAX = tax_table(whale_tax_cm)

whaledata.HWNM = sample_data(data.frame(whaleblow_var.migration.sh_Air.fil_HWNM, stringsAsFactors = F))

class(OTU.HWNM)

class(TAX)

class(whaledata.HWNM)

sample_names(OTU.HWNM)

sample_names(whaledata.HWNM)

## merge the files with phyloseq

whale1.HWNM = phyloseq(OTU.HWNM, TAX, whaledata.HWNM)

p.HWNM = ggrare(whale1.HWNM, step = 100, color = "Species", se = FALSE)

ggsave("rarefaction.curves.migration.Air.fil.HWNM.jpg", plot = p.HWNM , device = 'jpg', width = 168, height = 130, units = "mm",

dpi = 300, limitsize = TRUE)

##############

#HWSM

dat.migration.Air.fil.5_t.HWSM = dat.migration.Air.fil.5_t[,47:66]

whaleblow_var.migration.sh_Air.fil_HWSM = whaleblow_var.migration.sh_Air.fil_3[c(1:20),]

## they need to become matrix files to work with phyloseq

whale_otu_nm.HWSM = as.matrix(sapply(dat.migration.Air.fil.5_t.HWSM, as.numeric))

whale_tax_cm = as.matrix(sapply(attributes.migration_Silva.4, as.character))

## check they are matrices

class(whale_otu_nm.HWSM)

class(whale_tax_cm)

## convert files into phyloseq object types

OTU.HWSM = otu_table(whale_otu_nm.HWSM, taxa_are_rows = TRUE)

TAX = tax_table(whale_tax_cm)

whaledata.HWSM = sample_data(data.frame(whaleblow_var.migration.sh_Air.fil_HWSM, stringsAsFactors = F))

class(OTU.HWSM)

class(TAX)

class(whaledata.HWSM)

#phyloseq

sample_names(OTU.HWSM)

sample_names(whaledata.HWSM)

## merge the files with phyloseq

whale1.HWSM = phyloseq(OTU.HWSM, TAX, whaledata.HWSM)

p.HWSM = ggrare(whale1.HWSM, step = 100, color = "Species", se = FALSE)

ggsave("rarefaction.curves.migration.Air.fil.HWSM.jpg", plot = p.HWSM , device = 'jpg', width = 168, height = 130, units = "mm",

dpi = 300, limitsize = TRUE)

##############

#SWSM

dat.migration.Air.fil.5_t.SWSM = dat.migration.Air.fil.5_t[,67:73]

whaleblow_var.migration.sh_Air.fil_SWSM = whaleblow_var.migration.sh_Air.fil_3[c(21:27),]

## they need to become matrix files to work with phyloseq

whale_otu_nm.SWSM = as.matrix(sapply(dat.migration.Air.fil.5_t.SWSM, as.numeric))

whale_tax_cm = as.matrix(sapply(attributes.migration_Silva.4, as.character))

## check they are matrices

class(whale_otu_nm.SWSM)

class(whale_tax_cm)

## convert files into phyloseq object types

OTU.SWSM = otu_table(whale_otu_nm.SWSM, taxa_are_rows = TRUE)

TAX = tax_table(whale_tax_cm)

whaledata.SWSM = sample_data(data.frame(whaleblow_var.migration.sh_Air.fil_SWSM, stringsAsFactors = F))

class(OTU.SWSM)

class(TAX)

class(whaledata.SWSM)

#phyloseq

sample_names(OTU.SWSM)

sample_names(whaledata.SWSM)

## merge the files with phyloseq

whale1.SWSM = phyloseq(OTU.SWSM, TAX, whaledata.SWSM)

p.SWSM = ggrare(whale1.SWSM, step = 100, color = "Species", se = FALSE)

ggsave("rarefaction.curves.migration.Air.fil.SWSM.jpg", plot = p.SWSM , device = 'jpg', width = 168, height = 130, units = "mm",

dpi = 300, limitsize = TRUE)

##############

#SWNM

dat.migration.Air.fil.5_t.SWNM = dat.migration.Air.fil.5_t[,21:46]

whaleblow_var.migration.sh_Air.fil_SWNM = whaleblow_var.migration.sh_Air.fil_3[c(28:53),]

## they need to become matrix files to work with phyloseq

whale_otu_nm.SWNM = as.matrix(sapply(dat.migration.Air.fil.5_t.SWNM, as.numeric))

whale_tax_cm = as.matrix(sapply(attributes.migration_Silva.4, as.character))

## check they are matrices

class(whale_otu_nm.SWNM)

class(whale_tax_cm)

## convert files into phyloseq object types

OTU.SWNM = otu_table(whale_otu_nm.SWNM, taxa_are_rows = TRUE)

TAX = tax_table(whale_tax_cm)

whaledata.SWNM = sample_data(data.frame(whaleblow_var.migration.sh_Air.fil_SWNM, stringsAsFactors = F))

class(OTU.SWNM)

class(TAX)

class(whaledata.SWNM)

#phyloseq

sample_names(OTU.SWNM)

sample_names(whaledata.SWNM)

## merge the files with phyloseq

whale1.SWNM = phyloseq(OTU.SWNM, TAX, whaledata.SWNM)

p.SWNM = ggrare(whale1.SWNM, step = 100, color = 'species', se = FALSE)

ggsave("rarefaction.curves.migration.Air.fil.SWNM.jpg", plot = p.SWNM , device = 'jpg', width = 168, height = 130, units = "mm",

dpi = 300, limitsize = TRUE)

############################################

###Beta Diversity after Air.filtering

#Back to 'rel.abund.log' dataset: dat.migration.Air.fil.4.rel.abund

dat.migration.Air.fil.4.rel.abund.log = log(dat.migration.Air.fil.4.rel.abund +1)

#View(dat.migration.Air.fil.4.rel.abund.abund.log[,1:10])

dim(dat.migration.Air.fil.4.rel.abund.log )

# 73 8,037

#Bray-Curtis dissimilarity matrix with log

dat.migration.Air.fil.4.rel.abund.log.bc = vegdist(dat.migration.Air.fil.4.rel.abund.log, method = "bray")

#dendrogram

dat.migration.Air.fil.4.rel.abund.log.hclus = hclust(dat.migration.Air.fil.4.rel.abund.log.bc, method = "average")

cluster.diagram.Air.fil = plot(dat.migration.Air.fil.4.rel.abund.log.hclus)

ggsave("cluster.diagram.Air.fil .jpg", plot = cluster.diagram.Air.fil , device = 'jpg', width = 168, height = 100, units = "mm",

dpi = 300, limitsize = TRUE)

####nMDS

# nmds plot with dots of 2 colours (whale 'Species'), (stress should be <0.2)

dat.migration.Air.fil.4.rel.abund.log.hclus.mds = metaMDS(dat.migration.Air.fil.4.rel.abund.log.bc, autotransform = F, trace = F, trymax=50)

dat.migration.Air.fil.4.rel.abund.log.hclus.mds # stress = 0.1518477

#create plotdata

#change x and y to desired x and y axis names

plotData.migration.Air.fil = data.frame(dat.migration.Air.fil.4.rel.abund.log.hclus.mds$points, whaleblow_var.migration.sh_Air.fil_3$Species)

names(plotData.migration.Air.fil) = c("x","y","Species")

#plotData.migration.Air.fil.rem = plotData.migration.Air.fil[plotData.migration.Air.fil$x < 0.2,]

nMDS.Air.fil_ellipse =

ggplot(plotData.migration.Air.fil, aes(x,y,colour=Species, shape=Species)) + geom_point() +

theme_bw() +

theme(plot.title = element_text(size = 14, family = "Tahoma", face = "bold"),

text = element_text(size = 8, family = "Tahoma"),

axis.title = element_text(face="bold"),

axis.text.x=element_text(size = 11)) +

scale_fill_brewer(palette = "Accent") +

labs(x ="NMDS 1", y = "NMDS 2", fill = 'Species', face = 'bold') +

stat_ellipse()

ggsave("nMDS.Air.fil_ellipse.jpg", plot = nMDS.Air.fil_ellipse, device = 'jpg', width = 168, height = 100, units = "mm",

dpi = 300, limitsize = TRUE)

############################################

###Significance testing

#Use 'dat.migration.Air.fil.5' and whaleblow_var.migration.sh_Air.fil_3

dat.migration.Air.fil.5$Whale =rownames(dat.migration.Air.fil.5)

#Reorder dat.migration.counts.fil

dat.migration.Air.fil.5.2 = dat.migration.Air.fil.5[,c(8038,1:8037)]

View(dat.migration.Air.fil.4.2[,1:10])

View(dat.migration.Air.fil.4.2[,8030:8038])

dim(dat.migration.Air.fil.4.2)

#73 8038

#Join dat.migration.Air.fil.4.2 with whaleblow_var.migration.sh_Air.fil_3

dat.migration.Air.fil.5.comp = whaleblow_var.migration.sh_Air.fil_3 %>% left_join(dat.migration.Air.fil.5.2)

dim(dat.migration.Air.fil.5.comp)

#73 8039

###Now fit the generalised linear models.

OTU.Air.fil.binomial = mvabund(dat.migration.Air.fil.5.comp[,3:8038])

#create variable for log of total abundance of OTU's per sample

dat.migration.Air.fil.5.comp$logTotalAbundance = log(apply(dat.migration.Air.fil.5.comp[,3:8038],1,sum))

Pre.fit1.Air.fil = manyglm(OTU.Air.fil.binomial ~ dat.migration.Air.fil.5.comp$Species + offset(logTotalAbundance), data = dat.migration.Air.fil.5.comp, family="negative.binomial")

#check assumptions

plot(Pre.fit1.Air.fil)

# #This is the actual significance test

fit.1.Air.fil = manyglm(OTU.Air.fil.binomial ~ Species.migration.Air.fil +offset(logTotalAbundance), data = dat.migration.Air.fil.5.comp, family="negative.binomial")

fit.2.Air.fil = manyglm(OTU.Air.fil.binomial ~ offset(logTotalAbundance), data = dat.migration.Air.fil.5.comp, family="negative.binomial")

#adjusted

globalTest1.migration.Air.fil.adjusted = anova(fit.1.Air.fil, fit.2.Air.fil, nBoot=1000, p.uni='adjusted')

save(globalTest1.migration.Air.fil.adjusted, file='globalTest1.migration.Air.fil.adjusted.Rdata')

globalTest1_significant.binomial.Air.fil.adjusted = colnames(globalTest1.migration.Air.fil.adjusted$uni.p)[which(globalTest1.migration.Air.fil.adjusted$uni.p[2,]<0.05)]

############################################################################

#Which groups are different?

#creates a list to store results

anovas_pairwise_tests= list()

species_list = c("HumpbackSM","SeawaterSM","SeawaterNM","HumpbackNM")

Species = dat.migration.Air.fil.5.comp$Species

k=1

#loop through pairs of species

for (i in 1:3){

for (j in (i+1):4){

species1 = species_list[i]

species2 = species_list[j]

#subsets to only those species

dat_subset = subset(dat.migration.Air.fil.5.comp, Species %in% c(species1, species2))

dat_subset = subset(dat_subset , is.finite(logTotalAbundance))

dat_subset$Species = factor(dat_subset$Species)

OTUs = mvabund(select(dat_subset, -one_of(c("Whale","Species","logTotalAbundance"))))

#null and alternative hypotheses

fit.alternative = manyglm(OTUs~ Species +offset(logTotalAbundance), data=dat_subset)

fit.null = manyglm(OTUs~ offset(logTotalAbundance), data=dat_subset)

#anova.manyglm this does a likelihood ratio test. Takes a while depending on how many whales

#put the results into kth element of anovas_pairwise_tests

anovas_pairwise_tests [[k]] = anova(fit.null, fit.alternative, nBoot=1000)

#label the list so we can remember which pairwise test it was

names(anovas_pairwise_tests) [k] = paste("Unadjusted pvalue of anova for",species1,"vs",species2)

k=k+1

}

}

save(anovas_pairwise_tests,file='anovas_pairwise_tests.Rdata')

#load('anovas_pairwise_tests.Rdata')

##########################################################################

###Rel.abund. of taxa (zOTUs and Genus) of HWSM and HWNM

##dat.migration.Air.fil.5, whaleblow_var.migration.sh_ful.fil, attributes.migration_rdp

Whale.OTUs.Air.fil = names(dat.migration.Air.fil.5)

######Trim attributes.migration_rdp with Whale.OTUs.Air.fil

attributes.migration_rdp.2 = attributes.migration_rdp[,-8]

rownames(attributes.migration_rdp.2) = attributes.migration_rdp.2$variable

###Join attributes.migration_rdp_Air.fil with dat.migration.Air.fil.5

dat.migration.Air.fil.5_t = as.data.frame(t(dat.migration.Air.fil.5))

dat.migration.Air.fil.5_t$variable = rownames(dat.migration.Air.fil.5_t)

dat.migration.Air.fil.5_t.comp = dat.migration.Air.fil.5_t %>% left_join (attributes.migration_rdp.2)

dim(dat.migration.Air.fil.5_t.comp)

dat.migration.Air.fil.5_t.comp_2 = dat.migration.Air.fil.5_t.comp[,c(74:80,1:20,47:66)]

########################

###HW-SM

dat.migration.Air.fil.5_t.comp_2.HWSM = dat.migration.Air.fil.5_t.comp_2[,c(1:7,28:47)]

dat.migration.Air.fil.5_t.comp_2.HWSM_2 = dat.migration.Air.fil.5_t.comp_2.HWSM %>%

mutate(sum_of_rows = rowSums(dat.migration.Air.fil.5_t.comp_2.HWSM[,8:27]))

dat.migration.Air.fil.5_t.comp_2.HWSM_2.no0 = dat.migration.Air.fil.5_t.comp_2.HWSM_2 %>% filter (sum_of_rows > 0)

###

###zOTU

dat.migration.Air.fil.5_t.comp_2.HWSM_3 = dat.migration.Air.fil.5_t.comp_2.HWSM_2.no0 %>% mutate (Rel.abund.zOTU = sum_of_rows/sum(sum_of_rows))

Rel.abund.OTU.Air.fil.HWSM = dat.migration.Air.fil.5_t.comp_2.HWSM_3 [,c(1,29)]

mean(Rel.abund.OTU.Air.fil.HWSM$Rel.abund.zOTU)

median(Rel.abund.OTU.Air.fil.HWSM$Rel.abund.zOTU)

sd(Rel.abund.OTU.Air.fil.HWSM$Rel.abund.zOTU)

###

###Genus

#Extract col Genus and sum_of_rows

Rel.Abund.Genus.migration.Air.fil.HWSM = dat.migration.Air.fil.5_t.comp_2.HWSM_2[,c(7,28)]

Rel.Abund.Genus.migration.Air.fil.HWSM.2 = Rel.Abund.Genus.migration.Air.fil.HWSM %>% group_by(Genus) %>% summarise(sum_of_genus = sum(sum_of_rows)) %>% as.data.frame()

Rel.Abund.Genus.migration.Air.fil.HWSM.3 = Rel.Abund.Genus.migration.Air.fil.HWSM.2 %>% mutate (Rel.abund.Genus = sum_of_genus/sum(sum_of_genus))

#Bring in order

Rel.Abund.Genus.migration.Air.fil.HWSM.order =

Rel.Abund.Genus.migration.Air.fil.HWSM.3[order(Rel.Abund.Genus.migration.Air.fil.HWSM.3$Rel.abund.Genus),]

#Filter out 'unclassified'

Rel.Abund.Genus.migration.Air.fil.HWSM.order_2 = Rel.Abund.Genus.migration.Air.fil.HWSM.order %>% filter(!Genus == 'unclassified')

#Filter out '0'

Rel.Abund.Genus.migration.Air.fil.HWSM.order_3 = Rel.Abund.Genus.migration.Air.fil.HWSM.order_2 %>% filter(!Rel.abund.Genus == 0)

#Mean average of Rel.abund.Genus

mean(Rel.Abund.Genus.migration.Air.fil.HWSM.order_3$Rel.abund.Genus)

median(Rel.Abund.Genus.migration.Air.fil.HWSM.order_3$Rel.abund.Genus)

sd(Rel.Abund.Genus.migration.Air.fil.HWSM.order_3$Rel.abund.Genus)

########################

###HW-NM

dat.migration.Air.fil.5_t.comp_2.HWNM = dat.migration.Air.fil.5_t.comp_2[,c(1:27)]

dat.migration.Air.fil.5_t.comp_2.HWNM_2 = dat.migration.Air.fil.5_t.comp_2.HWNM %>%

mutate(sum_of_rows = rowSums(dat.migration.Air.fil.5_t.comp_2.HWNM[,8:27]))

dat.migration.Air.fil.5_t.comp_2.HWNM_2.no0 = dat.migration.Air.fil.5_t.comp_2.HWNM_2 %>% filter (sum_of_rows > 0)

###

###zOTU

dat.migration.Air.fil.5_t.comp_2.HWNM_3 = dat.migration.Air.fil.5_t.comp_2.HWNM_2.no0 %>% mutate (Rel.abund.zOTU = sum_of_rows/sum(sum_of_rows))

Rel.abund.OTU.Air.fil.HWNM = dat.migration.Air.fil.5_t.comp_2.HWNM_3 [,c(1,29)]

mean(Rel.abund.OTU.Air.fil.HWNM$Rel.abund.zOTU)

median(Rel.abund.OTU.Air.fil.HWNM$Rel.abund.zOTU)

sd(Rel.abund.OTU.Air.fil.HWNM$Rel.abund.zOTU)

###

###Genus

#Extract col Genus and sum_of_rows

Rel.Abund.Genus.migration.Air.fil.HWNM = dat.migration.Air.fil.5_t.comp_2.HWNM_2[,c(7,28)]

Rel.Abund.Genus.migration.Air.fil.HWNM.2 = Rel.Abund.Genus.migration.Air.fil.HWNM %>% group_by(Genus) %>% summarise(sum_of_genus = sum(sum_of_rows)) %>% as.data.frame()

Rel.Abund.Genus.migration.Air.fil.HWNM.3 = Rel.Abund.Genus.migration.Air.fil.HWNM.2 %>% mutate (Rel.abund.Genus = sum_of_genus/sum(sum_of_genus))

#Bring in order

Rel.Abund.Genus.migration.Air.fil.HWNM.order =

Rel.Abund.Genus.migration.Air.fil.HWNM.3[order(Rel.Abund.Genus.migration.Air.fil.HWNM.3$Rel.abund.Genus),]

#Filter out 'unclassified'

Rel.Abund.Genus.migration.Air.fil.HWNM.order_2 = Rel.Abund.Genus.migration.Air.fil.HWNM.order %>% filter(!Genus == 'unclassified')

#Filter out '0'

Rel.Abund.Genus.migration.Air.fil.HWNM.order_3 = Rel.Abund.Genus.migration.Air.fil.HWNM.order_2 %>% filter(!Rel.abund.Genus == 0)

#Mean average of Rel.abund.Genus

mean(Rel.Abund.Genus.migration.Air.fil.HWNM.order_3$Rel.abund.Genus)

median(Rel.Abund.Genus.migration.Air.fil.HWNM.order_3$Rel.abund.Genus)

sd(Rel.Abund.Genus.migration.Air.fil.HWNM.order_3$Rel.abund.Genus)

###################################################

###Relative abundance of classes (for class-level assignment)

#Join with attributes.migration_rdp.2

dat.migration.Air.fil.5_t.comp_10 =

dat.migration.Air.fil.5_t %>% left_join (attributes.migration_rdp.2)

#Reorder and only keep col Class

dat.migration.Air.fil.5_t.comp_Class = dat.migration.Air.fil.5_t.comp_10[,c(77,1:73)]

#Aggregate Class

dat.migration.Air.fil.5_t.comp_Class.aggr =

aggregate(dat.migration.Air.fil.5_t.comp_Class[,2:74],

by=list(Category=dat.migration.Air.fil.5_t.comp_Class$Class), FUN=sum)

#Turn col Class into rownames and delete col Class

dat.migration.Air.fil.5_t.comp_Class.aggr.2 = dat.migration.Air.fil.5_t.comp_Class.aggr

rownames(dat.migration.Air.fil.5_t.comp_Class.aggr.2) = dat.migration.Air.fil.5_t.comp_Class.aggr.2$Category

dat.migration.Air.fil.5_t.comp_Class.aggr.3 = dat.migration.Air.fil.5_t.comp_Class.aggr.2[,-1]

#Transpose dat.w16.counts.fil_t.comp.2.2.aggr.2

dat.migration.Air.fil.5_t.comp_Class.aggr.t =

as.data.frame(t(dat.migration.Air.fil.5_t.comp_Class.aggr.3))

##Turn dat.w16.counts.fil_t.comp.2.Class.2.t into rel.abund.

dat.migration.Air.fil.5_t.comp_Class.aggr.t.2 =

dat.migration.Air.fil.5_t.comp_Class.aggr.t %>%

mutate(sum_of_counts= rowSums(dat.migration.Air.fil.5_t.comp_Class.aggr.t))

dat.migration.Air.fil.5_t.comp_Class.aggr.t.2.rel.abund =

dat.migration.Air.fil.5_t.comp_Class.aggr.t.2[,1:45]/

dat.migration.Air.fil.5_t.comp_Class.aggr.t.2$sum_of_counts

#Give dat.migration.Air.fil.5_t.comp_Class.aggr.t.2.rel.abund rownames as column

dat.migration.Air.fil.5_t.comp_Class.aggr.t.2.rel.abund$Whale =

rownames(dat.migration.Air.fil.5_t.comp_Class.aggr.t)

Whale.Air.fil = dat.migration.Air.fil.5_t.comp_Class.aggr.t.2.rel.abund$Whale

Class_Wide.Air.fil = dat.migration.Air.fil.5_t.comp_Class.aggr.t.2.rel.abund

#Turn col 'Whale' into rownames and delete

rownames(Class_Wide.Air.fil) = Class_Wide.Air.fil$Whale

Class_Wide.Air.fil_2 = Class_Wide.Air.fil[,-46]

#Transpose Class_Wide_2

Class_Wide.Air.fil_t = as.data.frame(t(Class_Wide.Air.fil_2))

Classes = rownames(Class_Wide.Air.fil_t)

#Create col 'mean_of_rel.abund'

Class.mean = apply(Class_Wide.Air.fil_t,1,mean)

Class.sum = apply(Class_Wide.Air.fil_t,1,sum)

Class_Wide.Air.fil_t.2 = cbind(Class.mean,Class.sum,Class_Wide.Air.fil_t)

#Add Classes as col

Class_Wide.Air.fil_t.2$Class = Classes.Air.fil

#Reorder

Class_Wide.Air.fil_t.3 = Class_Wide.Air.fil_t.2[,c(76,1,3:75)]

#Filter out raw classes

Class_Wide.Air.fil_t.red = Class_Wide.Air.fil_t.3 %>% filter(Class.mean>=0.01)

#Turn col 'Class' into rownames

rownames(Class_Wide.Air.fil_t.red) = Class_Wide.Air.fil_t.red$Class

#Delete col 1 and 2

Class_Wide.Air.fil_t.red_2 = Class_Wide.Air.fil_t.red[,-c(1,2)]

####Create 'Class_Wide_t.other'

Class_Wide.Air.fil_t.other = Class_Wide.Air.fil_t.3 %>% filter(Class.mean<0.01)

rownames(Class_Wide.Air.fil_t.other) = Class_Wide.Air.fil_t.other$Class

Class_Wide.Air.fil_t.other_2 = Class_Wide.Air.fil_t.other[,-c(1,2)]

Class_Wide.Air.fil_t.other_2_t = as.data.frame(t(Class_Wide.Air.fil_t.other_2))

Class_Wide_t.other.sum = Class_Wide.Air.fil_t.other_2_t %>%

mutate(other=rowSums(Class_Wide.Air.fil_t.other_2_t))

Other = Class_Wide_t.other.sum[,36]

#Join Class_Wide.Air.fil_t.red_2 and Class_Wide.Air.fil_t.other_2

Class_Wide_t.mod = bind_rows(Class_Wide.Air.fil_t.red_2, Class_Wide.Air.fil_t.other_2)

#Transpose Class_Wide_t.red_2

Class_Wide.Air.fil_t.red_t = as.data.frame(t(Class_Wide.Air.fil_t.red_2))

#Convert rownames back to col 'Whale'

Class_Wide.Air.fil_t.red_t$Whale = rownames(Class_Wide.Air.fil_t.red_t)

#Join with 'Class.other'

Class_stacked = cbind(Class_Wide.Air.fil_t.red_t,Other)

#Reshape into long format

Barplot_Class_long = melt(Class_stacked, id.vars = "Whale", variable.name = "Class")

colnames(Barplot_Class_long)[3] = 'Relative.abundance.of.classes'

colourCount = length(unique(Barplot_Class_long$Class))

getPalette = colorRampPalette(brewer.pal(9, "Paired"))

Class_assignment =

ggplot(Barplot_Class_long, aes(x = Whale, y = Relative.abundance.of.classes, fill = Class)) +

geom_bar(stat = "identity") +

scale_fill_manual(values = getPalette(colourCount)) +

theme(axis.text.x=element_text(angle = -90),axis.text=element_text(size=5),#Whale names

axis.title=element_text(size=8,face="bold"),legend.text=element_text(size=6),#Class names

legend.title=element_text(size=8,face="bold"))

ggsave("Class_assignment.jpg", plot = Class_assignment, device = 'jpg', width = 168, height = 126, units = "mm",

dpi = 300, limitsize = TRUE)

###########################################################################

###Check genera of HWSM and HWNM for potential pathogens

#Rel.Abund.Genus.migration.Air.fil.HWSM.order_3

#Rel.Abund.Genus.migration.Air.fil.HWNM.order_3

pathogens.file = read.csv("Apprill.Supp.pathogens.csv",header=T, sep=',')

Genus.pathogens = pathogens.file$Genus

Genus.pathogens.2 = unique(Genus.pathogens)

###HWSM

Genus.HWSM = Rel.Abund.Genus.migration.Air.fil.HWSM.order_3$Genus

pathogens.HWSM = intersect(Genus.HWSM,Genus.pathogens.2)

rownames(Rel.Abund.Genus.migration.Air.fil.HWSM.order_3) = Rel.Abund.Genus.migration.Air.fil.HWSM.order_3$Genus

Genus.pathogens.HWSM.df = Rel.Abund.Genus.migration.Air.fil.HWSM.order_3 [pathogens.HWSM,]

sum(Genus.pathogens.HWSM.df$Rel.abund.Genus)

###HWNM

Genus.HWNM = Rel.Abund.Genus.migration.Air.fil.HWNM.order_3$Genus

pathogens.HWNM = intersect(Genus.HWNM,Genus.pathogens.2)

rownames(Rel.Abund.Genus.migration.Air.fil.HWNM.order_3) = Rel.Abund.Genus.migration.Air.fil.HWNM.order_3$Genus

Genus.pathogens.HWNM.df = Rel.Abund.Genus.migration.Air.fil.HWNM.order_3 [pathogens.HWNM,]

sum(Genus.pathogens.HWNM.df$Rel.abund.Genus)

setdiff(pathogens.HWSM,pathogens.HWNM)

pathogens.HW = c(pathogens.HWSM,pathogens.HWNM)

pathogens.HW.unique = unique(pathogens.HW)

##########################################################################

###Check Core zOTUs, Air.fil

####Core taxa

#Calculate core taxa HW-SM and HW-NM that 80% of individuals have in common

#dat.migration.Air.fil.5, whaleblow_var.migration.sh_ful.fil, attributes.migration_rdp

dat.migration.Air.fil.6 = dat.migration.Air.fil.5

dat.migration.Air.fil.6$Whale =rownames(dat.migration.Air.fil.6)

dat.migration.Air.fil.6.binary = dat.migration.Air.fil.6

#Convert dataframe into binary

dat.migration.Air.fil.6.binary [] = +(dat.migration.Air.fil.6.binary > 0)

#Transpose dat.migration.Air.fil.6.binary

dat.migration.Air.fil.6.binary_t = as.data.frame(t(dat.migration.Air.fil.6.binary))

####### ########## ############ ###############

###HumpbackSM

dat.migration.Air.fil.6.binary_t.HWSM = dat.migration.Air.fil.6.binary_t[,47:66]

#Create col 'Total.rel.abund.'

dat.migration.Air.fil.6.binary_t.HWSM.2 = dat.migration.Air.fil.6.binary_t.HWSM %>%

mutate (Total.rel.abund.OTU = rowSums(dat.migration.Air.fil.6.binary_t.HWSM)/20)

#Give dat.migration.Air.fil.6.binary_t.HWSM.2 rownames as col variable

dat.migration.Air.fil.6.binary_t.HWSM.2$variable = rownames(dat.migration.Air.fil.6.binary_t.HWSM)

#Reorder

dat.migration.Air.fil.6.binary_t.HWSM.3 = dat.migration.Air.fil.6.binary_t.HWSM.2[,c(21,22,1:20)]

#Join dat.migration.Air.fil.6.binary_t.HWSM.2 with attributes.migration_rdp

Core.zOTU.migration_HWSM.Air.fil = dat.migration.Air.fil.6.binary_t.HWSM.3 %>% left_join (attributes.migration_rdp)

#Reorder

Core.zOTU.migration_HWSM.Air.fil_2 = Core.zOTU.migration_HWSM.Air.fil[,c(1,2,28,3:22)]

names(Core.zOTU.migration_HWSM.Air.fil_2)

#Order according to Core.zOTU.migration_HWSM.Air.fil_2$Total.rel.abund.OTU

Core.zOTU.migration_HWSM.Air.fil_3 = Core.zOTU.migration_HWSM.Air.fil_2[order(Core.zOTU.migration_HWSM.Air.fil_2$Total.rel.abund.OTU),]

#Any coreOTUs above 80% (> 0.8)?

Core.zOTU.migration_HWSM.Air.fil_4_0.8 = Core.zOTU.migration_HWSM.Air.fil_3 %>% filter (Total.rel.abund.OTU >= 0.8)

Core.OTU.migration.Air.fil.HWSM_rel.abund = Core.zOTU.migration_HWSM.Air.fil_4_0.8 %>% left_join(Rel.abund.OTU.Air.fil.HWSM)

###

#CoreGenus

Core.Genus.migration_HWSM.Air.fil_2 = Core.zOTU.migration_HWSM.Air.fil[,c(28,27,3:22)]

names(Core.Genus.migration_HWSM.Air.fil_2)

Core.Genus.migration_HWSM.Air.fil_3 = Core.Genus.migration_HWSM.Air.fil_2 [order(Core.Genus.migration_HWSM.Air.fil_2$Genus),]

Core.Genus.migration_HWSM.Air.fil_4 = aggregate(Core.Genus.migration_HWSM.Air.fil_3[,3:22], by=list(Category = Core.Genus.migration_HWSM.Air.fil_3$Genus), FUN=sum)

#Save col 1 as vector (Genus.all.migration.HWSM)

Genus.all.migration.HWSM.Air.fil = Core.Genus.migration_HWSM.Air.fil_4$Category

#Delete col 1 from Genus.OTU.migration.HWSM_3.Air.fil

Core.Genus.migration_HWSM.Air.fil_5 = Core.Genus.migration_HWSM.Air.fil_4[,-c(1)]

#Convert dataframe into binary

Core.Genus.migration_HWSM.Air.fil_5[] = +(Core.Genus.migration_HWSM.Air.fil_5 > 0)

#Create col 'Total.rel.abund'

Core.Genus.migration_HWSM.Air.fil_6 = Core.Genus.migration_HWSM.Air.fil_5 %>% mutate (Total.rel.abund.Genus = rowSums(Core.Genus.migration_HWSM.Air.fil_5[])/20)

#Join Core.Genus.migration_HWSM.Air.fil_6 with Genus.all.migration.HWSM again

Core.Genus.migration.HWSM.Air.fil_7 = cbind(Genus.all.migration.HWSM.Air.fil, Core.Genus.migration_HWSM.Air.fil_6)

Core.Genus.migration.HWSM.Air.fil_8 = Core.Genus.migration.HWSM.Air.fil_7 [order(Core.Genus.migration.HWSM.Air.fil_7$Total.rel.abund.Genus),]

Core.Genus.migration.HWSM.Air.fil_9 = Core.Genus.migration.HWSM.Air.fil_8 [,c(1,22)]

#Any core genera above 80% (> 0.8)?

Core.Genus.migration.HWSM.Air.fil_10 = Core.Genus.migration.HWSM.Air.fil_9 %>% filter (Total.rel.abund.Genus >= 0.8)

#Rename col 1 of Core.Genus.migration.HWSM.Air.fil_10

colnames(Core.Genus.migration.HWSM.Air.fil_10)[1] = "Genus"

Core.Genus.migration.Air.fil.HWSM.rel.abund = Core.Genus.migration.HWSM.Air.fil_10 %>% left_join(Rel.Abund.Genus.migration.Air.fil.HWSM.order_2, by='Genus')

####### ########## ############ ###############

###HumpbackNM

dat.migration.Air.fil.6.binary_t.HWNM = dat.migration.Air.fil.6.binary_t[,1:20]

#Create col 'Total.rel.abund.'

dat.migration.Air.fil.6.binary_t.HWNM.2 = dat.migration.Air.fil.6.binary_t.HWNM %>%

mutate (Total.rel.abund.OTU = rowSums(dat.migration.Air.fil.6.binary_t.HWNM)/20)

#Give dat.migration.Air.fil.6.binary_t.HWNM.2 rownames as col variable

dat.migration.Air.fil.6.binary_t.HWNM.2$variable = rownames(dat.migration.Air.fil.6.binary_t.HWNM)

#Reorder

dat.migration.Air.fil.6.binary_t.HWNM.3 = dat.migration.Air.fil.6.binary_t.HWNM.2[,c(21,22,1:20)]

#Join dat.migration.Air.fil.6.binary_t.HWNM.2 with attributes.migration_rdp

Core.zOTU.migration_HWNM.Air.fil = dat.migration.Air.fil.6.binary_t.HWNM.3 %>% left_join (attributes.migration_rdp)

#Reorder

Core.zOTU.migration_HWNM.Air.fil_2 = Core.zOTU.migration_HWNM.Air.fil[,c(1,2,28,3:22)]

names(Core.zOTU.migration_HWNM.Air.fil_2)

#Order according to Core.zOTU.migration_HWNM.Air.fil_2$Total.rel.abund.OTU

Core.zOTU.migration_HWNM.Air.fil_3 = Core.zOTU.migration_HWNM.Air.fil_2[order(Core.zOTU.migration_HWNM.Air.fil_2$Total.rel.abund.OTU),]

#Any coreOTUs above 80% (> 0.8)?

Core.zOTU.migration_HWNM.Air.fil_4_0.8 = Core.zOTU.migration_HWNM.Air.fil_3 %>% filter (Total.rel.abund.OTU >= 0.8)

Core.OTU.Air.fil.HWNM_rel.abund = Core.zOTU.migration_HWNM.Air.fil_4_0.8 %>% left_join(Rel.abund.OTU.Air.fil.HWNM)

###

#CoreGenus

Core.Genus.migration_HWNM.Air.fil_2 = Core.zOTU.migration_HWNM.Air.fil[,c(28,27,3:22)]

names(Core.Genus.migration_HWNM.Air.fil_2)

Core.Genus.migration_HWNM.Air.fil_3 = Core.Genus.migration_HWNM.Air.fil_2 [order(Core.Genus.migration_HWNM.Air.fil_2$Genus),]

Core.Genus.migration_HWNM.Air.fil_4 = aggregate(Core.Genus.migration_HWNM.Air.fil_3[,3:22], by=list(Category = Core.Genus.migration_HWNM.Air.fil_3$Genus), FUN=sum)

#Save col 1 as vector (Genus.all.migration.HWNM)

Genus.all.migration.HWNM.Air.fil = Core.Genus.migration_HWNM.Air.fil_4$Category

#Delete col 1 from Genus.OTU.migration.HWNM_3

Core.Genus.migration_HWNM.Air.fil_5 = Core.Genus.migration_HWNM.Air.fil_4[,-c(1)]

#Convert dataframe into binary

Core.Genus.migration_HWNM.Air.fil_5[] = +(Core.Genus.migration_HWNM.Air.fil_5 > 0)

#Create col 'Total.rel.abund.'

Core.Genus.migration_HWNM.Air.fil_6 = Core.Genus.migration_HWNM.Air.fil_5 %>% mutate (Total.rel.abund.Genus = rowSums(Core.Genus.migration_HWNM.Air.fil_5[])/20)

#Join Core.Genus.migration_HWNM.Air.fil_6 with Genus.all.migration.HWNM.Air.fil again

Core.Genus.migration.HWNM_7 = cbind(Genus.all.migration.HWNM.Air.fil, Core.Genus.migration_HWNM.Air.fil_6)

Core.Genus.migration.HWNM_8 = Core.Genus.migration.HWNM_7 [order(Core.Genus.migration.HWNM_7$Total.rel.abund.Genus),]

Core.Genus.migration.HWNM_9 = Core.Genus.migration.HWNM_8 [,c(1,22)]

#Any coregenera above 80% (> 0.8)?

Core.Genus.migration.HWNM_10 = Core.Genus.migration.HWNM_9 %>% filter (Total.rel.abund.Genus >= 0.8)

#Rename col 1 of Core.Genus.migration.HWNM_10

colnames(Core.Genus.migration.HWNM_10)[1] = "Genus"

Core.Genus.migration.Air.fil.HWNM.rel.abund = Core.Genus.migration.HWNM_10 %>% left_join(Rel.Abund.Genus.migration.Air.fil.HWNM.order_3, by='Genus')

######## ########### ############# #################

######## ########### ############# #################

###Significance testing --> Test the two whale groups to see which zOTUs are significantly different

#Use 'dat.migration.Air.fil.5' and whaleblow_var.migration.sh_Air.fil_3

#Trim whaleblow_var.migration.sh_Air.fil_3 accordingly (get rid of seawater)

whaleblow_var.migration.sh_Air.fil_HW = whaleblow_var.migration.sh_Air.fil_3[c(1:20,54:73),]

#Trim dat.migration.Air.fil.5 accordingly (get rid of seawater)

dat.migration.Air.fil.HW = dat.migration.Air.fil.5[c(1:20, 47:66),]

#Filter out zOTUs that equal zero!

dat.migration.Air.fil.HW_t = as.data.frame(t(dat.migration.Air.fil.HW))

dat.migration.Air.fil.HW_t.2 = dat.migration.Air.fil.HW_t

dat.migration.Air.fil.HW_t.2$variable = rownames(dat.migration.Air.fil.HW_t.2)

dim(dat.migration.Air.fil.HW_t.2)

#8037 41

dat.migration.Air.fil.HW_t.3 = dat.migration.Air.fil.HW_t.2 %>% mutate (sum_of_rows = rowSums(dat.migration.Air.fil.HW_t.2[,1:40]))

dat.migration.Air.fil.HW_t.no0 = dat.migration.Air.fil.HW_t.3 %>% filter (!sum_of_rows == 0)

rownames(dat.migration.Air.fil.HW_t.no0) = dat.migration.Air.fil.HW_t.no0$variable

dat.migration.Air.fil.HW_t.no0 = dat.migration.Air.fil.HW_t.no0[,-c(41,42)]

dat.migration.Air.fil.HW_t.no0.t = as.data.frame((t(dat.migration.Air.fil.HW_t.no0)))

dat.migration.Air.fil.HW_t.no0.t$Whale = rownames(dat.migration.Air.fil.HW_t.no0.t)

#Reorder dat.migration.Air.fil.HW_t.no0.t

dat.migration.Air.fil.HW_t.no0.t.2 = dat.migration.Air.fil.HW_t.no0.t[,c(6089,1:6088)]

dat.migration.Air.fil.HW_3 = dat.migration.Air.fil.HW_t.no0.t.2

#Join dat.migration.Air.fil.HW.3 with whaleblow_var.migration.sh_Air.fil_HW

dat.migration.Air.fil.HW.comp = whaleblow_var.migration.sh_Air.fil_HW %>% left_join(dat.migration.Air.fil.HW_3)

###Now fit the generalised linear models.

OTU.Air.fil.binomial.HW = mvabund(dat.migration.Air.fil.HW.comp[,3:6090])

#create variable for log of total abundance of OTU's per sample

dat.migration.Air.fil.HW.comp$logTotalAbundance = log(apply(dat.migration.Air.fil.HW.comp[,3:6090],1,sum))

Pre.fit1.Air.fil = manyglm(OTU.Air.fil.binomial.HW ~ Species + offset(logTotalAbundance), data = dat.migration.Air.fil.HW.comp, family="negative.binomial")

#check assumptions

plot(Pre.fit1.Air.fil)

# #This is the actual significance test

fit.1.Air.fil.HW = manyglm(OTU.Air.fil.binomial.HW ~ Species +offset(logTotalAbundance), data = dat.migration.Air.fil.HW.comp)

fit.2.Air.fil.HW = manyglm(OTU.Air.fil.binomial.HW ~ offset(logTotalAbundance), data = dat.migration.Air.fil.HW.comp)

#

# #adjusted

globalTest1.migration.Air.fil.HW.adjusted = anova(fit.1.Air.fil.HW, fit.2.Air.fil.HW, nBoot=1000, p.uni='adjusted')

save(globalTest1.migration.Air.fil.HW.adjusted, file='globalTest1.migration.Air.fil.HW.adjusted.Rdata')

globalTest1_significant.binomial.Air.fil.adjusted.HW = colnames(globalTest1.migration.Air.fil.HW.adjusted$uni.p)[which(globalTest1.migration.Air.fil.HW.adjusted$uni.p[2,]<0.05)]

##################################################################################

###Check out these globalTest1_significant.binomial.Air.fil.unadjusted.HW

#Delete row whale

Core.zOTU.migration_HWNM.Air.fil_4_0.8_2 = Core.zOTU.migration_HWNM.Air.fil_4_0.8[-69,]

Core.zOTU.migration_HWNM.Air.fil= Core.zOTU.migration_HWNM.Air.fil_4_0.8_2$variable

#Do sign.zOTus.HW and core.zOTUs have any zOTUs in common?

intersect(Core.zOTU.migration_HWNM.Air.fil, globalTest1_significant.binomial.Air.fil.unadjusted.HW)

###Which taxa do these sign.zOTUs belong to?

attributes.migration_rdp.10 = attributes.migration_rdp

rownames(attributes.migration_rdp.10) = attributes.migration_rdp.10$variable

attributes.migration_rdp.sign.zOTUs.HW = attributes.migration_rdp.10[globalTest1_significant.binomial.Air.fil.unadjusted.HW,]

View(attributes.migration_rdp.sign.zOTUs.HW)

attributes.migration_rdp.sign.zOTUs.HW.order = attributes.migration_rdp.sign.zOTUs.HW[order(attributes.migration_rdp.sign.zOTUs.HW$Genus),]

View(attributes.migration_rdp.sign.zOTUs.HW.order)

####HWNM

#Are all these sign. zOTUs present in HWNM?

length(intersect(attributes.migration_rdp.sign.zOTUs.HW.order$variable, Rel.abund.OTU.Air.fil.HWNM$variable))

#Join with Rel.abund.OTU.Air.fil.HWNM to add rel. abund.

attributes.migration_rdp.sign.zOTUs.HW.order_HWNM = attributes.migration_rdp.sign.zOTUs.HW.order %>% left_join (Rel.abund.OTU.Air.fil.HWNM)

#Aggregate on Genus

attributes.migration_rdp.sign.zOTUs.HW.order_HWNM.genus.sum = attributes.migration_rdp.sign.zOTUs.HW.order_HWNM[,c(7,9)]

attributes.migration_rdp.sign.zOTUs.HW.order_HWNM.genus.sum.2 =

aggregate(attributes.migration_rdp.sign.zOTUs.HW.order_HWNM.genus.sum$Rel.abund.zOTU,

list(attributes.migration_rdp.sign.zOTUs.HW.order_HWNM.genus.sum$Genus),FUN=sum)

attributes.migration_rdp.sign.zOTUs.HW.order_HWNM.genus.sum.2.order =

attributes.migration_rdp.sign.zOTUs.HW.order_HWNM.genus.sum.2[order(attributes.migration_rdp.sign.zOTUs.HW.order_HWNM.genus.sum.2$x),]

attributes.migration_rdp.sign.zOTUs.HW.order_HWNM.genus.sum.2.order.2 = attributes.migration_rdp.sign.zOTUs.HW.order_HWNM.genus.sum.2.order[-17,]

#Total relative abundance of these significant zOTUs:

sum(attributes.migration_rdp.sign.zOTUs.HW.order_HWNM.genus.sum.2.order.2$x)

#0.3273504

####HWSM

#Are all these sign. zOTUs present in HWSM?

length(intersect(attributes.migration_rdp.sign.zOTUs.HW.order$variable, Rel.abund.OTU.Air.fil.HWSM$variable))

#Join with Rel.abund.OTU.Air.fil.HWSM to add rel. abund.

attributes.migration_rdp.sign.zOTUs.HW.order_HWSM = attributes.migration_rdp.sign.zOTUs.HW.order %>% left_join (Rel.abund.OTU.Air.fil.HWSM)

#Aggregate on Genus

attributes.migration_rdp.sign.zOTUs.HW.order_HWSM.genus.sum = attributes.migration_rdp.sign.zOTUs.HW.order_HWSM[,c(7,9)]

attributes.migration_rdp.sign.zOTUs.HW.order_HWSM.genus.sum.2 =

attributes.migration_rdp.sign.zOTUs.HW.order_HWSM.genus.sum %>% filter(!Rel.abund.zOTU == 'NA')

attributes.migration_rdp.sign.zOTUs.HW.order_HWSM.genus.sum.3 =

aggregate(attributes.migration_rdp.sign.zOTUs.HW.order_HWSM.genus.sum.2$Rel.abund.zOTU,

list(attributes.migration_rdp.sign.zOTUs.HW.order_HWSM.genus.sum.2$Genus),FUN=sum)

attributes.migration_rdp.sign.zOTUs.HW.order_HWSM.genus.sum.order =

attributes.migration_rdp.sign.zOTUs.HW.order_HWSM.genus.sum.3[order(attributes.migration_rdp.sign.zOTUs.HW.order_HWSM.genus.sum.3$x),]

#Which genera are shared?

intersect(attributes.migration_rdp.sign.zOTUs.HW.order_HWSM.genus.sum.order$Group.1, attributes.migration_rdp.sign.zOTUs.HW.order_HWNM.genus.sum.2.order.2$Group.1)

##################################################################################

###Create heatmap with those significantly different zOTUs (HW-SM vs. HW-NM)

#Transpose

dat.migration.Air.fil.HW_t.no0_t = as.data.frame(t(dat.migration.Air.fil.HW_t.no0))

#Rel.abund.

dat.migration.Air.fil.HW_t.no0_t.rel.abund = dat.migration.Air.fil.HW_t.no0_t/rowSums(dat.migration.Air.fil.HW_t.no0_t)

dat.migration.Air.fil.HW_t.no0_t.rel.abund.sign = dat.migration.Air.fil.HW_t.no0_t.rel.abund[,globalTest1_significant.binomial.Air.fil.unadjusted.HW]

#Transpose

dat.migration.Air.fil.HW_t.no0_t.rel.abund.sign_t = as.data.frame(t(dat.migration.Air.fil.HW_t.no0_t.rel.abund.sign))

dim(dat.migration.Air.fil.HW_t.no0_t.rel.abund.sign_t)

###Reduce dataset to 50 most abundant zOTUs

###Determine mean rel.abund. of zOTUs

#Convert rownames into col

dat.migration.Air.fil.HW_t.no0_t.rel.abund.sign_t.2 = dat.migration.Air.fil.HW_t.no0_t.rel.abund.sign_t

dat.migration.Air.fil.HW_t.no0_t.rel.abund.sign_t.2$variable = rownames(dat.migration.Air.fil.HW_t.no0_t.rel.abund.sign_t.2)

#Determine mean rel.abund. of zOTUs by creating col mean.rel.abund

dat.migration.Air.fil.HW_t.no0_t.rel.abund.sign_t.3 = dat.migration.Air.fil.HW_t.no0_t.rel.abund.sign_t.2 %>%

mutate (mean.rel.abund = rowMeans(dat.migration.Air.fil.HW_t.no0_t.rel.abund.sign_t.2[,1:40]))

#Give dat.migration.Air.fil.HW_t.no0_t.rel.abund.sign_t.3 rownames back and delete col variable

rownames(dat.migration.Air.fil.HW_t.no0_t.rel.abund.sign_t.3) = dat.migration.Air.fil.HW_t.no0_t.rel.abund.sign_t.3$variable

#Order dat.migration.Air.fil.HW_t.no0_t.rel.abund.sign_t.3 after mean.rel.abund

dat.migration.Air.fil.HW_t.no0_t.rel.abund.sign_t.4 =

dat.migration.Air.fil.HW_t.no0_t.rel.abund.sign_t.3[order(-dat.migration.Air.fil.HW_t.no0_t.rel.abund.sign_t.3[,42]),]

###Choose the first 50 most abundant zOTUs

dat.migration.Air.fil.HW_t.no0_t.rel.abund.sign_t.4_50 = dat.migration.Air.fil.HW_t.no0_t.rel.abund.sign_t.4[1:50,]

#Delete last two cols (variable and mean.rel.abund)

dat.migration.Air.fil.HW_t.no0_t.rel.abund.sign_t.4_50.2 = dat.migration.Air.fil.HW_t.no0_t.rel.abund.sign_t.4_50[,-c(41,42)]

rownames(whaleblow_var.migration.sh_Air.fil_HW) = whaleblow_var.migration.sh_Air.fil_HW$Whale

whaleblow_var.migration.sh_Air.fil_HW.10 = whaleblow_var.migration.sh_Air.fil_HW [-2]

#Create heatmap

pheatmap(dat.migration.Air.fil.HW_t.no0_t.rel.abund.sign_t.4_50.2, annotation_col = whaleblow_var.migration.sh_Air.fil_HW.10,

clustering_distance_rows = "manhattan",clustering_distance_cols = "manhattan", cluster_cols = FALSE, clustering_method = 'average')

#log transformation

dat.migration.Air.fil.HW_t.no0_t.rel.abund.sign_t.4_50.2.log = log10(dat.migration.Air.fil.HW_t.no0_t.rel.abund.sign_t.4_50.2+1)

################################Sqrt transformation

#forth-sqrt transformation

dat.migration.Air.fil.HW_t.no0_t.rel.abund.sign_t.4_50.2.matrix = data.matrix(dat.migration.Air.fil.HW_t.no0_t.rel.abund.sign_t.4_50.2)

dat.migration.Air.fil.HW_t.no0_t.rel.abund.sign_t.4_50.2.matrix.4sqrt = nthroot(dat.migration.Air.fil.HW_t.no0_t.rel.abund.sign_t.4_50.2.matrix, 4)

###Add taxonomy to zOTUs

#Change rownames (Add taxa)

rownames.heatmap.HW = c('Zotu681_Geobacillus','Zotu7_Oceanospirillales','Zotu31_Fusobacteriales', 'Zotu33_Flavobacteriaceae', 'Zotu163_Leptotrichiaceae',

'Zotu64_Leptotrichiaceae', 'Zotu378_Gammaproteobacteria', 'Zotu26_Arcobacter', 'Zotu65_Guggenheimella', 'Zotu105_Helcococcus',

'Zotu134_Bacteroidetes', 'Zotu41_Leptotrichiaceae', 'Zotu155_Flavobacteriaceae', 'Zotu3570_Proteobacteria','Zotu184_Flavobacteriaceae',

'Zotu189_Gammaproteobacteria', 'Zotu15_Flavobacteriaceae', 'Zotu371_Pelomonas', 'Zotu12_Flavobacteriaceae', 'Zotu230_Candidatus_Pelagibacter',

'Zotu171_Helcococcus', 'Zotu74_Corynebacterium', 'Zotu52_Corynebacterium', 'Zotu48_Bacteria', 'Zotu62_Rhodobacteraceae', 'Zotu109_Gammaproteobacteria',

'Zotu969_Arcobacter', 'Zotu181_Tenacibaculum', 'Zotu45_Moraxellaceae', 'Zotu684_Corynebacterium', 'Zotu34_Arcobacter', 'Zotu16_Bacteria',

'Zotu75_Enhydrobacter', 'Zotu754_Gammaproteobacteria', 'Zotu83_Bacteroidetes', 'Zotu78_Gammaproteobacteria', 'Zotu71_Leptotrichiaceae',

'Zotu55_Bacteria', 'Zotu187_Microbacteriaceae', 'Zotu47_Leptotrichiaceae', 'Zotu177_Helcococcus', 'Zotu2919_Mycoplasma', 'Zotu77_Gammaproteobacteria',

'Zotu39_Bacteroidetes', 'Zotu56_Candidatus_Pelagibacter', 'Zotu88_Bacteria', 'Zotu288_Candidatus_Pelagibacter', 'Zotu107_Gammaproteobacteria',

'Zotu106_Bacteroidetes', 'Zotu188_Helcococcus')

dat.migration.Air.fil.HW_t.no0_t.rel.abund.sign_t.4_50.2.matrix.4sqrt.df = as.data.frame(dat.migration.Air.fil.HW_t.no0_t.rel.abund.sign_t.4_50.2.matrix.4sqrt)

rownames.heatmap.HW.df = as.data.frame(rownames.heatmap.HW)

dat.migration.Air.fil.HW_t.no0_t.rel.abund.sign_t.4_50.2.matrix.4sqrt_2 = bind_cols(rownames.heatmap.HW.df,dat.migration.Air.fil.HW_t.no0_t.rel.abund.sign_t.4_50.2.matrix.4sqrt.df)

rownames(dat.migration.Air.fil.HW_t.no0_t.rel.abund.sign_t.4_50.2.matrix.4sqrt_2) = dat.migration.Air.fil.HW_t.no0_t.rel.abund.sign_t.4_50.2.matrix.4sqrt_2$rownames.heatmap.HW

dat.migration.Air.fil.HW_t.no0_t.rel.abund.sign_t.4_50.2.matrix.4sqrt_3 = dat.migration.Air.fil.HW_t.no0_t.rel.abund.sign_t.4_50.2.matrix.4sqrt_2[,-1]

dat.migration.Air.fil.5.rel.abund.sign_t.3_50.2.log.cc =

pheatmap(dat.migration.Air.fil.HW_t.no0_t.rel.abund.sign_t.4_50.2.matrix.4sqrt_3, annotation_col = whaleblow_var.migration.sh_Air.fil_HW.10,

clustering_distance_rows = "manhattan", fontsize_row = 4,

fontsize_col = 4,

clustering_distance_cols = "manhattan", cluster_cols = FALSE, clustering_method = 'average')

whaleblow_var.migration.sh_Air.fil_HW.10.ch = apply(whaleblow_var.migration.sh_Air.fil_HW.10, 1, FUN = as.character)

whaleblow_var.migration.sh_Air.fil_HW.10.ch.2 = as.data.frame(whaleblow_var.migration.sh_Air.fil_HW.10.ch)

names(whaleblow_var.migration.sh_Air.fil_HW.10.ch.2)[1] = 'Species'

# Assign colours to groups:

SpeciesCol.after = c("forestgreen", "blue3")

names(SpeciesCol.after) = levels(whaleblow_var.migration.sh_Air.fil_HW.10.ch.2$Species)

# Add to a list, where names match those in factors dataframe

AnnColour.after = list(Species = SpeciesCol.after)

# Check the output

AnnColour.after

dat.migration.HW.rel.abund.sign_50.4sqrt.cc =

pheatmap(dat.migration.Air.fil.HW_t.no0_t.rel.abund.sign_t.4_50.2.matrix.4sqrt_3, annotation_col =

whaleblow_var.migration.sh_Air.fil_HW.10.ch.2, clustering_distance_rows = "manhattan", fontsize = 4, fontsize_row = 3,

fontsize_col = 3, angle_col = '45',legend_labels = 2, color = colorRampPalette(rev(brewer.pal(n = 9, name ="Blues")))(100),

clustering_distance_cols = "manhattan", cluster_cols = FALSE, clustering_method = 'average',

annotation_colors = AnnColour.after)

ggsave("heatmap.HW.sign.4sqrt.jpg", plot = dat.migration.HW.rel.abund.sign_50.4sqrt.cc, device = 'jpg', width = 168, height = 80, units = "mm",

dpi = 300, limitsize = TRUE)

#################################################################

###Heatmap after filtering: heatmap of significant OTUs of seawater and whales

## Use 'dat.migration.Air.fil.5' and globalTest1_significant.binomial.Air.fil.adjusted, whaleblow_var.migration.sh_Air.fil_3

#Rel.abund.

dat.migration.Air.fil.5.rel.abund = dat.migration.Air.fil.5/rowSums(dat.migration.Air.fil.5)

dat.migration.Air.fil.5.rel.abund.sign = dat.migration.Air.fil.5.rel.abund[,globalTest1_significant.binomial.Air.fil.adjusted]

#Transpose

dat.migration.Air.fil.5.rel.abund.sign_t = as.data.frame(t(dat.migration.Air.fil.5.rel.abund.sign))

###Reduce dataset to 50 most abundant zOTUs

###Determine mean rel.abund. of zOTUs

#Convert rownames into col

dat.migration.Air.fil.5.rel.abund.sign_t$variable = rownames(dat.migration.Air.fil.5.rel.abund.sign_t)

#Determine mean rel.abund. of zOTUs by creating col mean.rel.abund

dat.migration.Air.fil.5.rel.abund.sign_t.2 = dat.migration.Air.fil.5.rel.abund.sign_t %>%

mutate (mean.rel.abund = rowMeans(dat.migration.Air.fil.5.rel.abund.sign_t[,1:73]))

#Give dat.migration.Air.fil.5.rel.abund.sign_t.2 rownames back and delete col variable

rownames(dat.migration.Air.fil.5.rel.abund.sign_t.2) = dat.migration.Air.fil.5.rel.abund.sign_t.2$variable

#Order dat.migration.Air.fil.5.rel.abund.sign_t.2 after mean.rel.abund

dat.migration.Air.fil.5.rel.abund.sign_t.3 =

dat.migration.Air.fil.5.rel.abund.sign_t.2[order(-dat.migration.Air.fil.5.rel.abund.sign_t.2[,75]),]

###Choose the first 50 most abundant zOTUs

dat.migration.Air.fil.5.rel.abund.sign_t.3_50 = dat.migration.Air.fil.5.rel.abund.sign_t.3[1:50,]

#Delete last two cols (variable and mean.rel.abund)

dat.migration.Air.fil.5.rel.abund.sign_t.3_50.2 = dat.migration.Air.fil.5.rel.abund.sign_t.3_50[,-c(74,75)]

rownames(whaleblow_var.migration.sh_Air.fil_3) = whaleblow_var.migration.sh_Air.fil_3$Whale

whaleblow_var.migration.sh_Air.fil_4 = whaleblow_var.migration.sh_Air.fil_3 [-2]

#Create heatmap

pheatmap(dat.migration.Air.fil.5.rel.abund.sign_t.3_50.2, annotation_col = whaleblow_var.migration.sh_Air.fil_4,

clustering_distance_rows = "manhattan",clustering_distance_cols = "manhattan", cluster_cols = FALSE, clustering_method = 'average')

write.csv(dat.migration.Air.fil.5.rel.abund.sign_t.3_50.2.log,file='dat.migration.Air.fil.5.rel.abund.sign_t.3_50.2.log.csv')

#log transformation

dat.migration.Air.fil.5.rel.abund.sign_t.3_50.2.log = log10(dat.migration.Air.fil.5.rel.abund.sign_t.3_50.2+1)

###Add taxonomy to zOTUs

#Change rownames (Add taxa)

rownames.heatmap.Air.fil = c("Zotu23_Gammaproteobacteria", "Zotu28_Candidatus_Pelagibacter", "Zotu12_Flavobacteriaceae", "Zotu29_Bacteria", "Zotu8_Enhydrobacter", "Zotu35_Rhodobacteraceae",

"Zotu16_Bacteria","Zotu24_Bacteria","Zotu681_Geobacillus", "Zotu44_Arcobacter", "Zotu7_Oceanospirillales", "Zotu120_Rhodobacteraceae", "Zotu69_Bacteria",

"Zotu40_Bacteria", "Zotu153_Rhodobacteraceae", "Zotu51_Marinimicrobia", "Zotu118_Flavobacteriaceae", "Zotu141_Candidatus_Pelagibacter",

"Zotu197_Polaribacter", "Zotu86_Cyanobacteria", "Zotu53_Alphaproteobacteria", "Zotu943_Bradyrhizobium", "Zotu1752_Rubrobacter", "Zotu113_Candidatus_Pelagibacter",

"Zotu126_Bacteria", "Zotu584_Gammaproteobacteria", "Zotu168_Cyanobacteria", "Zotu130_Alphaproteobacteria", "Zotu411_Flavobacteriaceae", "Zotu77_Gammaproteobacteria",

"Zotu215_Candidatus_Pelagibacter", "Zotu239_Candidatus_Pelagibacter", "Zotu160_Candidatus_Pelagibacter", "Zotu18_Gammaproteobacteria", "Zotu33_Flavobacteriaceae",

"Zotu46_Gammaproteobacteria", "Zotu2260_Candidatus_Pelagibacter", "Zotu102", "Zotu492_Alphaproteobacteria", "Zotu637_Gammaproteobacteria", "Zotu17_Flavobacteriaceae",

"Zotu76_Microbacteriaceae", "Zotu107_Gammaproteobacteria", "Zotu30_Cardiobacteriales", "Zotu363_Candidatus_Pelagibacter", "Zotu129_Bacteria", "Zotu38_Flavobacteriaceae",

"Zotu378_Gammaproteobacteria", "Zotu98_Gammaproteobacteria", "Zotu405_Proteobacteria")

rownames.heatmap.Air.fil.df = as.data.frame(rownames.heatmap.Air.fil)

dat.migration.Air.fil.5.rel.abund.sign_t.3_50.2.log_2 = bind_cols(rownames.heatmap.Air.fil.df,dat.migration.Air.fil.5.rel.abund.sign_t.3_50.2.log)

rownames(dat.migration.Air.fil.5.rel.abund.sign_t.3_50.2.log_2) = dat.migration.Air.fil.5.rel.abund.sign_t.3_50.2.log_2$rownames.heatmap.Air.fil

dat.migration.Air.fil.5.rel.abund.sign_t.3_50.2.log_3 = dat.migration.Air.fil.5.rel.abund.sign_t.3_50.2.log_2[,-1]

dat.migration.Air.fil.5.rel.abund.sign_t.3_50.2.log.cc = pheatmap(dat.migration.Air.fil.5.rel.abund.sign_t.3_50.2.log_3, annotation_col = whaleblow_var.migration.sh_Air.fil_4,

clustering_distance_rows = "manhattan", fontsize = 4, fontsize_row = 3,

fontsize_col = 3, angle_col = '45',legend_labels = 2,

clustering_distance_cols = "manhattan", cluster_cols = FALSE, clustering_method = 'average')

ggsave("heatmap.Air.fil.log.jpg", plot = dat.migration.Air.fil.5.rel.abund.sign_t.3_50.2.log.cc, device = 'jpg', width = 168, height = 80, units = "mm",

dpi = 300, limitsize = TRUE)

################################Sqrt transformation

#forth-sqrt transformation

dat.migration.Air.fil.5.rel.abund.sign_t.3_50.2.matrix = data.matrix(dat.migration.Air.fil.5.rel.abund.sign_t.3_50.2)

dat.migration.Air.fil.5.rel.abund.sign_t.3_50.2.matrix.4sqrt = nthroot(dat.migration.Air.fil.5.rel.abund.sign_t.3_50.2.matrix, 4)

###Add taxonomy to zOTUs

#Change rownames (Add taxa)

rownames.heatmap.Air.fil = c("Zotu23_Gammaproteobacteria", "Zotu28_Candidatus_Pelagibacter", "Zotu12_Flavobacteriaceae", "Zotu29_Bacteria", "Zotu8_Enhydrobacter", "Zotu35_Rhodobacteraceae",

"Zotu16_Bacteria","Zotu24_Bacteria","Zotu681_Geobacillus", "Zotu44_Arcobacter", "Zotu7_Oceanospirillales", "Zotu120_Rhodobacteraceae", "Zotu69_Bacteria",

"Zotu40_Bacteria", "Zotu153_Rhodobacteraceae", "Zotu51_Marinimicrobia", "Zotu118_Flavobacteriaceae", "Zotu141_Candidatus_Pelagibacter",

"Zotu197_Polaribacter", "Zotu86_Cyanobacteria", "Zotu53_Alphaproteobacteria", "Zotu943_Bradyrhizobium", "Zotu1752_Rubrobacter", "Zotu113_Candidatus_Pelagibacter",

"Zotu126_Bacteria", "Zotu584_Gammaproteobacteria", "Zotu168_Cyanobacteria", "Zotu130_Alphaproteobacteria", "Zotu411_Flavobacteriaceae", "Zotu77_Gammaproteobacteria",

"Zotu215_Candidatus_Pelagibacter", "Zotu239_Candidatus_Pelagibacter", "Zotu160_Candidatus_Pelagibacter", "Zotu18_Gammaproteobacteria", "Zotu33_Flavobacteriaceae",

"Zotu46_Gammaproteobacteria", "Zotu2260_Candidatus_Pelagibacter", "Zotu102_Candidatus_Pelagibacter", "Zotu492_Alphaproteobacteria", "Zotu637_Gammaproteobacteria", "Zotu17_Flavobacteriaceae",

"Zotu76_Microbacteriaceae", "Zotu107_Gammaproteobacteria", "Zotu30_Cardiobacteriales", "Zotu363_Candidatus_Pelagibacter", "Zotu129_Bacteria", "Zotu38_Flavobacteriaceae",

"Zotu378_Gammaproteobacteria", "Zotu98_Gammaproteobacteria", "Zotu405_Proteobacteria")

dat.migration.Air.fil.5.rel.abund.sign_t.3_50.2.4sqrt.df = as.data.frame(dat.migration.Air.fil.5.rel.abund.sign_t.3_50.2.matrix.4sqrt)

rownames.heatmap.Air.fil.df = as.data.frame(rownames.heatmap.Air.fil)

dat.migration.Air.fil.5.rel.abund.sign_t.3_50.2.4sqrt_2 = bind_cols(rownames.heatmap.Air.fil.df,dat.migration.Air.fil.5.rel.abund.sign_t.3_50.2.4sqrt.df)

rownames(dat.migration.Air.fil.5.rel.abund.sign_t.3_50.2.4sqrt_2) = dat.migration.Air.fil.5.rel.abund.sign_t.3_50.2.4sqrt_2$rownames.heatmap.Air.fil

dat.migration.Air.fil.5.rel.abund.sign_t.3_50.2.4sqrt_3 = dat.migration.Air.fil.5.rel.abund.sign_t.3_50.2.4sqrt_2[,-1]

dat.migration.Air.fil.5.rel.abund.sign_t.3_50.2.log.cc =

pheatmap(dat.migration.Air.fil.5.rel.abund.sign_t.3_50.2.4sqrt_3, annotation_col = whaleblow_var.migration.sh_Air.fil_4,

clustering_distance_rows = "manhattan", fontsize_row = 4,

fontsize_col = 4,

clustering_distance_cols = "manhattan", cluster_cols = FALSE, clustering_method = 'average')

whaleblow_var.migration.sh_Air.fil_4.ch = apply(whaleblow_var.migration.sh_Air.fil_4, 1, FUN = as.character)

whaleblow_var.migration.sh_Air.fil_4.ch.2 = as.data.frame(whaleblow_var.migration.sh_Air.fil_4.ch)

names(whaleblow_var.migration.sh_Air.fil_4.ch.2)[1] = 'Species'

# Assign colours to groups:

SpeciesCol.after = c("forestgreen", "blue3", "grey80", "red")

names(SpeciesCol.after) = levels(whaleblow_var.migration.sh_Air.fil_4.ch.2$Species)

# Add to a list, where names match those in factors dataframe

AnnColour.after = list(Species = SpeciesCol.after)

# Check the output

dat.migration.Air.fil.5.rel.abund.sign_t.3_50.2.4sqrt.cc = pheatmap(dat.migration.Air.fil.5.rel.abund.sign_t.3_50.2.4sqrt_3, annotation_col =

whaleblow_var.migration.sh_Air.fil_4.ch.2, clustering_distance_rows = "manhattan", fontsize = 4, fontsize_row = 3,

fontsize_col = 3, angle_col = '45',legend_labels = 2, color = colorRampPalette(rev(brewer.pal(n = 9, name ="Blues")))(100),

clustering_distance_cols = "manhattan", cluster_cols = FALSE, clustering_method = 'average',

annotation_colors = AnnColour.after)

ggsave("heatmap.Air.fil.sign.4sqrt.jpg", plot = dat.migration.Air.fil.5.rel.abund.sign_t.3_50.2.4sqrt.cc, device = 'jpg', width = 168, height = 80, units = "mm",

dpi = 300, limitsize = TRUE)

##########################################################################

###UniFrac distances

#Delete col 74 (variable)

dat.migration.Air.fil.5_t.2 = dat.migration.Air.fil.5_t[,-74]

#Smallest number of reads?

min(colSums(dat.migration.Air.fil.5_t.2))

#3432

dat.migration.Air.fil.20 = dat.migration.Air.fil.5[,-8038]

otu_table_red2 = dat.migration.Air.fil.20

write.csv(otu_table_red2, 'otu_table_red2.csv')

#Rarefy to min(colSums(dat.migration.Air.fil.5_t.2)) --> 3432

otu_table_rff = rrarefy(x = otu_table_red2, sample = 3432)

#Read fasta file with otu sequences

otu_fasta = read.fasta(file = 'Sequences.of.zOTUs.fasta', as.string = T)

str(otu_fasta)

#List of 9572

otu_table_red = dat.migration.Air.fil.5_t.2

write.csv(otu_table_red, 'otu_table_red.csv')

#Filter reads

otu_ids = rownames(otu_table_red)

fasta_out = file(description = 'otus_red.fasta', open = 'w')

for(id in otu_ids)

{

write(x = paste('>', id, sep = ''), file = fasta_out)

write(x = as.character(otu_fasta[which(names(otu_fasta) == id)]), file = fasta_out)

}

close(fasta_out)

# Windows

# Alignment with mafft

system("WinUser/mafft.bat --auto otus_red.fasta > otus_red.aln")

# Tree with Fasttree

shell("WinUser\\FastTree.exe -nt otus_red.aln > otus_red.tre")

# Read the newly generated tree

otu_tree = read.tree('otus_red.tre')

plot(otu_tree)

is.rooted(otu_tree)

#FALSE

############################################################################

###Potential pathogens in HumpbackNM and HumpbackSM

#Delete col whale (col 8038)

dat.migration.Air.fil.50 = dat.migration.Air.fil.5[,-8038]

dim(dat.migration.Air.fil.50)

# 73 8037

#Rel.abund

dat.migration.Air.fil.4.rel.abund =

dat.migration.Air.fil.5/rowSums(dat.migration.Air.fil.5)

##################

###Only look at HumpbackSM zOTUs now

dat.migration.Air.fil.HWSM = dat.migration.Air.fil.50[47:66,]

#Transpose

dat.migration.Air.fil.HWSM_t = as.data.frame(t(dat.migration.Air.fil.HWSM))

dat.migration.Air.fil.HWSM_t.2 =

dat.migration.Air.fil.HWSM_t[which(rowSums(dat.migration.Air.fil.HWSM_t) > 1),]

#Turn rownames into col

dat.migration.Air.fil.HWSM_t.3 = dat.migration.Air.fil.HWSM_t.2

dat.migration.Air.fil.HWSM_t.3$variable = rownames(dat.migration.Air.fil.HWSM_t.3)

#Combine with attributes.migration_rdp

dat.migration.Air.fil.HWSM_t.4 = left_join(dat.migration.Air.fil.HWSM_t.3,

attributes.migration_rdp)

###How many of the zOTUs have UNclassified genera?

dat.migration.Air.fil.HWSM_t.5 = dat.migration.Air.fil.HWSM_t.4 %>%

filter(Genus == 'unclassified')

###Delete unclassified genera from dataset.

dat.migration.Air.fil.HWSM_t.6 = dat.migration.Air.fil.HWSM_t.4 %>%

filter(!Genus == 'unclassified')

#Delete cols that are not needed

dat.migration.Air.fil.HWSM_t.7 = dat.migration.Air.fil.HWSM_t.6[,c(21,26,27)]

Genera_HWSM_unique = unique(dat.migration.Air.fil.HWSM_t.7$Genus)

#Read Apprill.Supp.pathogens.csv

Apprill.Supp.pathogens = read.csv('Apprill.Supp.pathogens.csv')

Apprill.Supp.pathogens.unique = unique(Apprill.Supp.pathogens$Genus)

Potential.pathos.HWSM = intersect(Genera_HWSM_unique,Apprill.Supp.pathogens.unique)

###Sort out only bacteria that are potential marine mammal pathogens

Apprill.Supp.pathogens.MarineM = Apprill.Supp.pathogens %>% filter(!Previous.marine.mammal.occurrence.and.possible.connection.to.health.condition == 'not described')

Apprill.Supp.pathogens.MarineM.unique = unique(Apprill.Supp.pathogens.MarineM$Genus)

#How many potential marine mammal pathogens do HumpbackSM carry

Potential.pathos.MM.HWSM = intersect(Genera_HWSM_unique,Apprill.Supp.pathogens.MarineM.unique)

########################################################

###What is the relative abundance of those genera?

#Transpose dat.migration.Air.fil.HWSM_t.2

dat.migration.Air.fil.HWSM_t.t = as.data.frame(t(dat.migration.Air.fil.HWSM_t.2))

dat.migration.Air.fil.HWSM_t.10 = dat.migration.Air.fil.HWSM_t.4[,c(21:28,1:20)]

dat.migration.Air.fil.HWSM_t.11 = dat.migration.Air.fil.HWSM_t.10 %>%

mutate(sum_of_rows = rowSums(dat.migration.Air.fil.HWSM_t.10[,9:28]))

#Relative abundance of zOTUs

dat.migration.Air.fil.HWSM_t.12 = dat.migration.Air.fil.HWSM_t.11 %>%

mutate (Rel.abund.zOTU = sum_of_rows/sum(sum_of_rows))

###Relative abundance of genera

dat.migration.Air.fil.HWSM_t.13 = dat.migration.Air.fil.HWSM_t.12[,c(7,29)]

dat.migration.Air.fil.HWSM_t.14 = dat.migration.Air.fil.HWSM_t.13 %>% group_by(Genus) %>% summarise(sum_of_genus = sum(sum_of_rows)) %>% as.data.frame()

dat.migration.Air.fil.HWSM_t.15 = dat.migration.Air.fil.HWSM_t.14 %>% mutate (Rel.abund.Genus = sum_of_genus/sum(sum_of_genus))

#Bring in order

dat.migration.Air.fil.HWSM_t.15.order =

dat.migration.Air.fil.HWSM_t.15[order(dat.migration.Air.fil.HWSM_t.15$Rel.abund.Genus),]

#View(dat.migration.Air.fil.HWSM_t.15.order)

dat.migration.Air.fil.HWSM_t.15.order.2 = dat.migration.Air.fil.HWSM_t.15.order

rownames(dat.migration.Air.fil.HWSM_t.15.order.2) = dat.migration.Air.fil.HWSM_t.15.order.2$Genus

###Only keep potential pathogens in dat.migration.Air.fil.HWSM_t.15.order.2

#General potential pathogens

dat.migration.Air.fil.HWSM_t.15.GP = dat.migration.Air.fil.HWSM_t.15.order.2[Potential.pathos.HWSM,]

sum(dat.migration.Air.fil.HWSM_t.15.GP$Rel.abund.Genus)

#0.2101738

#Marine mammal pathogens

#Exclude Arthrobacter and Burkholderia, as they are not found to cause disease in marine mammals.

dat.migration.Air.fil.HWSM_t.15.MM = dat.migration.Air.fil.HWSM_t.15.order.2[Potential.pathos.MM.HWSM,]

dat.migration.Air.fil.HWSM_t.15.MM.2 = dat.migration.Air.fil.HWSM_t.15.MM[-c(4,10),]

##################

###Only look at HumpbackNM zOTUs now

dat.migration.Air.fil.HWNM = dat.migration.Air.fil.50[1:20,]

#Transpose

dat.migration.Air.fil.HWNM_t = as.data.frame(t(dat.migration.Air.fil.HWNM))

dat.migration.Air.fil.HWNM_t.2 =

dat.migration.Air.fil.HWNM_t[which(rowSums(dat.migration.Air.fil.HWNM_t) > 1),]

#Turn rownames into col

dat.migration.Air.fil.HWNM_t.3 = dat.migration.Air.fil.HWNM_t.2

dat.migration.Air.fil.HWNM_t.3$variable = rownames(dat.migration.Air.fil.HWNM_t.3)

#Combine with attributes.migration_rdp

dat.migration.Air.fil.HWNM_t.4 = left_join(dat.migration.Air.fil.HWNM_t.3,

attributes.migration_rdp)

###How many of the zOTUs have UNclassified genera?

dat.migration.Air.fil.HWNM_t.5 = dat.migration.Air.fil.HWNM_t.4 %>%

filter(Genus == 'unclassified')

###Delete unclassified genera from dataset.

dat.migration.Air.fil.HWNM_t.6 = dat.migration.Air.fil.HWNM_t.4 %>%

filter(!Genus == 'unclassified')

#Delete cols that are not needed

dat.migration.Air.fil.HWNM_t.7 = dat.migration.Air.fil.HWNM_t.6[,c(21,26,27)]

#How many different genera are present?

length(unique(dat.migration.Air.fil.HWNM_t.7$Genus))

Genera_HWNM_unique = unique(dat.migration.Air.fil.HWNM_t.7$Genus)

Apprill.Supp.pathogens.unique = unique(Apprill.Supp.pathogens$Genus)

Potential.pathos.HWNM = intersect(Genera_HWNM_unique,Apprill.Supp.pathogens.unique)

length(Potential.pathos.HWNM)

###Sort out only bacteria that are potential marine mammal pathogens

length(unique(Apprill.Supp.pathogens.MarineM$Genus))

Apprill.Supp.pathogens.MarineM.unique = unique(Apprill.Supp.pathogens.MarineM$Genus)

#How many potential marine mammal pathogens do HumpbackNM carry

Potential.pathos.MM.HWNM = intersect(Genera_HWNM_unique,Apprill.Supp.pathogens.MarineM.unique)

length(Potential.pathos.MM.HWNM)
